# Dietary α-Eleostearic Acid Ameliorates Experimental Inflammatory Bowel Disease in Mice by Activating Peroxisome Proliferator-Activated Receptor-γ

**DOI:** 10.1371/journal.pone.0024031

**Published:** 2011-08-31

**Authors:** Stephanie N. Lewis, Lera Brannan, Amir J. Guri, Pinyi Lu, Raquel Hontecillas, Josep Bassaganya-Riera, David R. Bevan

**Affiliations:** 1 Genetics, Bioinformatics, and Computational Biology Program, Virginia Tech, Blacksburg, Virginia, United States of America; 2 Department of Biochemistry, Virginia Tech, Blacksburg, Virginia, United States of America; 3 Nutritional Immunology and Molecular Medicine Laboratory, Virginia Bioinformatics Institute, Blacksburg, Virginia, United States of America; 4 Center for Modeling Immunology to Enteric Pathogens, Virginia Bioinformatics Institute, Blacksburg, Virginia, United States of America; Ulm University, Germany

## Abstract

**Background:**

Treatments for inflammatory bowel disease (IBD) are modestly effective and associated with side effects from prolonged use. As there is no known cure for IBD, alternative therapeutic options are needed. Peroxisome proliferator-activated receptor-gamma (PPARγ) has been identified as a potential target for novel therapeutics against IBD. For this project, compounds were screened to identify naturally occurring PPARγ agonists as a means to identify novel anti-inflammatory therapeutics for experimental assessment of efficacy.

**Methodology/Principal Findings:**

Here we provide complementary computational and experimental methods to efficiently screen for PPARγ agonists and demonstrate amelioration of experimental IBD in mice, respectively. Computational docking as part of virtual screening (VS) was used to test binding between a total of eighty-one compounds and PPARγ. The test compounds included known agonists, known inactive compounds, derivatives and stereoisomers of known agonists with unknown activity, and conjugated trienes. The compound identified through VS as possessing the most favorable docked pose was used as the test compound for experimental work. With our combined methods, we have identified α-eleostearic acid (ESA) as a natural PPARγ agonist. Results of ligand-binding assays complemented the screening prediction. In addition, ESA decreased macrophage infiltration and significantly impeded the progression of IBD-related phenotypes through both PPARγ-dependent and –independent mechanisms in mice with experimental IBD.

**Conclusions/Significance:**

This study serves as the first significant step toward a large-scale VS protocol for natural PPARγ agonist screening that includes a massively diverse ligand library and structures that represent multiple known target pharmacophores.

## Introduction

Inflammatory bowel disease (IBD) is a chronic and recurring inflammatory disease with two clinical manifestations: ulcerative colitis (UC) and Crohn's disease (CD). UC and CD affect over 4 million Americans and accrue a significant portion of the estimated $1.7 billion in health care costs for prevalent gastrointestinal diseases (CDC2007). While the etiopathogenesis of IBD remains unclear, it has been suggested that chronic mucosal inflammation characteristic of IBD is associated with a disruption in immune homeostasis [1]. As such, treatments for IBD should correct this immune dysregulation in order to prevent or reduce gut mucosal damage.

There is no cure for IBD, but treatments are available to combat the associated symptoms. One such treatment, 5-aminosalicylic acid, targets the nuclear hormone receptor peroxisome proliferator-activated receptor-gamma (PPARγ), which is highly expressed in the colonic epithelial and immune cells [2]–[7]. PPARγ and PPARδ serve as targets for the treatment of inflammatory and immune-mediated diseases because of the role they play in maintaining homeostasis and suppressing inflammation [1], [7]–[9]. PPARγ in particular is known to play a role in transcriptional regulation of anti-inflammatory processes via co-activator recruitment [6], [9], [10]. Ligand-induced activation of PPARγ can antagonize the activity of pro-inflammatory transcription factors such as nuclear factor kappa-light-chain-enhancer of activated B cells (NF-κB), signal transducer and activator of transcription (STAT), and activator protein (AP)-1 [11]. Other IBD treatments currently available include infliximab, which is an anti-tumor necrosis factor-alpha (TNF-α) antibody [12], [13], and corticosteroids, which systemically suppress immunity [14]. These medications are modestly successful for the long-term management of IBD but are associated with significant side effects, including increased risk of infection and cancer [15], [16]. Interestingly, the insulin-sensitizing PPARγ agonists used for treating type 2 diabetes, such as rosiglitazone and pioglitazone, have proven useful at ameliorating IBD effects in humans with UC [17]. However, rosiglitazone, and other PPARγ agonists of the thiazolidinediones (TZD) class of anti-diabetic drugs, are unlikely to be adopted by gastroenterologists for the treatment of IBD due to associated side effects [17] including hepatotoxicity, weight gain, fluid retention leading to edema, and congestive heart failure [18]. In this regard, the U.S. Food and Drug Administration (FDA) restricted the use of rosiglitazone in 2010 due to its side effects, whereas the European Medicines Agency completely banned its use in the European market. Natural therapeutics, such as fatty acids that induce PPARγ activation, might be a safer alternative to current treatments and TZDs.

Our group has conducted several preclinical animal model studies to suggest that supplementation of diet with fatty acids, such as conjugated linoleic acid (CLA) [8], [19] or agonistic botanicals, is effective at ameliorating colonic inflammation in mouse and pig models of IBD through a PPARγ-dependent mechanism [8], [19]–[21]. In an effort to expedite the drug and natural product therapeutic discovery process, virtual screening (VS) can complement traditional experimental methods for identification of novel PPARγ agonists. VS represents a cost- and time-efficient means of screening thousands of compounds within thematic libraries that justify further experimental assessment [22]. We are undertaking VS to identify novel PPARγ agonists within a collective of large compound databases. As a feasibility test, we screened a small group of known and proposed agonists, with the inclusion of known negative controls. The focus of this small-scale screen was to test our PPARγ structural model, and assess binding of natural compounds, with significant emphasis on conjugated trienes.

Conjugated trienes were selected due in part to their structural similarity to CLA. In addition, conjugated trienes exhibit effectiveness at ameliorating chronic inflammation [23], [24]. One such compound, α-eleostearic acid (ESA; 9Z11E13E-18:3), has been found at concentrations of 60–80% in tung and bitter gourd seed oils [25]. ESA has been shown to suppress tumor angiogenesis [26] and MCF-7 breast cancer cell proliferation via PPARγ activation [27], induce apoptosis via lipid peroxidation [28], and induce autophagy-dependent cell death through AKT/mTOR and ERK1/2 signal targeting [29]. Evidence also indicates that punicic acid plays a significant role in increasing lipid peroxidation [30] and inhibiting TNF-α-induced neutrophil hyperactivation to protect against experimentally induced colon inflammation in rats [31]. Our group has found that punicic acid ameliorates type 2 diabetes-induced inflammation by activating PPARγ and PPARα, and repressing TNF-α expression in white adipose tissue and liver [24] and increases peripheral insulin sensitivity [32] without causing any adverse side effects [33]. We have also demonstrated that punicic acid prevents experimental IBD through PPARγ- and PPARδ-dependent mechanisms [34]. Catalpic acid improves abdominal fat deposition, improves glucose homeostasis and up-regulates PPARα expression in adipose tissue of mice [23]. Though these plant-derived conjugated trienes suggest anti-inflammatory efficacy in various disease models, it has been suggested that ESA induces a greater degree of antioxidant activity than punicic acid in mice [35]. Punicic acid ameliorates both diabetes [34] and gut inflammation [24] without causing side effects [33], whereas ESA elicits mainly anti-inflammatory and anti-carcinogenic effects [26]–[29]. A goal of this study was to test the effectiveness of ESA in an experimental IBD model. Additionally, small-scale VS was conducted to test the predictability of our VS protocol for identifying PPARγ full agonists in the hopes of finding natural therapeutics and/or prophylactics for treating IBD and other chronic inflammation-related diseases. The computational portion of our study revealed information complementary to the predictions of our *in vitro* analysis, pre-clinical efficacy, and mechanistic testing in mice.

## Methods

### Docking procedure

AutoDock 4.0 [36] (AD4) was used for structural model testing, while AutoDock Vina [37] (Vina) was used for screening a subset of our in-house ligand database against the selected structural models of PPARγ. AutoDock Tools 1.5.2 (ADT) was used to build the appropriate charged protein and ligand files for docking. Default values for the Lamarckian Genetic Algorithm (LGA) were used for docking with AD4, with the exception of the maximum number of energy evaluations, which was reduced to 250,000. Adjusting this number reduced the screening time without significantly affecting pose prediction. Five iterations of AD4 with 50 poses generated per iteration were conducted for the re-docking step totaling 250 poses per protein structure model. Vina was used for cross-docking and to run the small-scale screening. Three Vina iterations were conducted for each ligand in the cross-docking step, while a single run was conducted for the small-scale screening. As a means to further sample conjugated triene geometry, three AD4 iterations of 50 poses each were run for each compound, which was a total of 150 poses per conjugated triene for each selected protein structure model. Scripts available through the AD4 development site (http://autodock/scripps.edu/) were modified and used to automate the screening process. Modifications to the scripts included exchanging the AD4 executable for the Vina executable and all subsequent necessary changes for Vina functionality.

### Structural Model Selection: Re-docking component

Five structures with co-crystallized rosiglitazone were downloaded from the Research Collaboratory for Structural Bioinformatics (RCSB) Protein Data Bank (PDB) [38], [39] (http://www.pdb.org). The selected structure IDs were 1FM6 [40], 1ZGY [41], 2PRG [42], 3CS8 [43], and 3DZY [44]. These structures were evaluated to identify a PPARγ structural model that would be appropriate for docking in a full agonist-like pose. Completeness of structure, crystal resolution, and re-docking ability were the factors considered. Re-docking refers to the ability of a docking program to reproduce the co-crystallized binding geometry and orientation of the associated ligand given a rigid macromolecule state. The PDB structures were superimposed and rosiglitazone was isolated from each protein structural model with the UCSF Chimera software package [45].

Re-docking was conducted with both native and non-native initial rosiglitazone conformations. Native refers to use of coordinates for the co-crystallized ligand structure of the respective protein structure model, whereas non-native refers to use of initial coordinates not found in the original PDB file. For the native test, each isolated rosiglitazone was re-docked into its respective protein structure (e.g., five protein models each with a different rosiglitazone coordinate files). For the non-native test, a single rosiglitazone structure was randomly selected for re-docking into all five structure models. Ligand flexibility and random initial geometry for the ligand reduced possible bias associated with use of a native ligand for one test structure, which was non-native for the other four. A comparison of results for the native and non-native ligand re-docking suggested the randomized initial conformation for rosiglitazone does not affect pose prediction as the predicted poses for both test sets were similar (data not shown). The non-native procedure involved docking of a single ligand structure to the protein structures, which is similar to what would be used for large-scale screening. Therefore, data from the non-native re-docking was analyzed and provided here. Both the superimposed positioning and the use of a single rosiglitazone model established a relatively controlled test set: overlaid coordinate space for the test structures, which translated to similar grid areas, with a single ligand coordinate file for testing.

### Structural Model Selection: Cross-docking component

Co-crystallized ligands from various PDB files were used for cross-docking to test predictability for other known agonists. Cross-docking refers to docking different ligand structures isolated from multiple PDB structures of the same protein to a single selected model structure. Ligands from 1FM9 [40], 2F4B [46], 2HWQ [47], 2I4J [48], 2I4P [48], 2VSR [49], 2VST [49], 3ET3 [50], 2VV0 [49], 2VV1 [49], 2ZK1 [51], and 2ZK2 [51] were included in the library for this purpose (Table 3).

### Small-scale in-house ligand library construction

Our small-scale ligand library included the rosiglitazone structure from re-docking, several of the cross-docking ligands, known PPARγ agonists, and known inactive compounds. Inclusion of the ligands from the re-docking and cross-docking steps served as controls for successful and unsuccessful docking. A search of published literature was conducted to find both naturally and synthetically derived compounds shown experimentally to either activate or not activate PPARγ [52]–[55]. Structural models for non-crystallized ligands were downloaded from the UCSF ZINC database online (http://zinc.docking.org/). Any structures not available through ZINC were built using the Dundee PRODRG2 server [56] (http://davapc1.bioch.dundee.ac.uk/prodrg/). Structures built with PRODRG2 were examined to ensure conservation of stereochemistry. Charges for all of the ligands in the database and the protein were generated using ADT. Eighty-one compounds total were tested in this study. A complete list of ligands included in the test library can be found in Table S1.

### Docking analysis for re-docking and cross-docking

The most energetically favorable pose for each ligand of the re-docking (25 lowest energy poses) and cross-docking (108 lowest energy poses) steps were used for analysis. Reference poses for root mean-squared deviation (RMSD) calculations were taken from crystal structure complexes for each ligand. These protein-ligand complex structures were superimposed onto the test structures to obtain a common coordinate space prior to the RMSD calculation. For re-docking, RMSD values are exact given each PPARγ-rosiglitazone complex was used as the reference for the respective results. However, the reported RMSD values for cross-docking were relative rather than absolute given the co-crystallized reference ligand coordinates are not relative to the protein structure models used for testing. The idea of relative RMSD stems from differences in side chain rotamers between the crystal structures. Side chain position is governed, in part, by ligand binding, which meant differences could be seen in binding cavity residue positions when the rosiglitazone-bound test structures were compared to each additional PPARγ structure model. These differences, which affect intramolecular interactions, resulted in minor deviations of the backbone on some regions for the superimposed structures relative to the test structure. This could mean the position of each co-crystallized reference ligand relative to the test structures was shifted slightly as well. However, there were areas of the backbone that superimposed without noticeable deviations. As the deviations between backbone positions were not consistent, adjusting for any rotamer-induced shifts in co-crystallized ligand coordinates was not feasible. Therefore, RMSD values for docked poses for each ligand were deemed “relative” as an acknowledgement of these minor variations in coordinates. An average RMSD, population standard deviation, and variance were calculated for each ligand (See Formulas S1). Re-docking and cross-docking results for each ligand relative to each test protein structure were deemed successful if the RMSD was less than 2.0 Å [57].

Docking success versus failure for re-docking and cross-docking was assessed qualitatively as well. Docked poses for rosiglitazone on the surface of the protein or near the opening of the binding cavity were deemed unsuccessful. Poses for which the molecule was not properly oriented, such as the imidizole ring of rosiglitazone positioned near the cavity opening rather than near the rear of the pocket, were deemed unsuccessful as well given such orientations would not match the co-crystallized coordinates. Similar conditions relative to each cross-docking ligand were also identified and assessed.

### Docking analysis for small-scale VS

To prepare for analysis of the small-scale VS results, interactions from various crystal structures were identified and cataloged. Reported crystal structure interactions for the five rosiglitazone-containing structures from the re-docking step and six fatty acid-containing structures from the cross-docking step were compiled using RCSB Ligand Explorer [58]. Residue atoms common to more than one interaction list for a specific ligand type were pooled and used as a reference list for analysis after docking. As such, there were two master interaction lists: rosiglitazone-like interactions (Table S2) and fatty acid-like interactions (Table S3). Common interactions between the two lists were also noted (Table S4).

Perl [59] scripts to automate pose distance measurement calculations and pose interaction predictions were also composed and used. The most energetically favorable docked pose for each ligand relative to the macromolecule were pooled for analysis. Only the potential for a ligand to fall into the full agonist category of ligands was assessed in depth for this study. Full agonism has been suggested to require interactions with Ser289, His323, His449, and Tyr473, which are residues positioned in the portion of the binding cavity proximal to the activation function-two (AF-2) region (Figure S1). Interactions in this region govern AF-2 conformational changes necessary for PPARγ activation. Distance measurements between the top docked poses (77 lowest energy poses) were calculated and used to predict interactions. Interactions similar to those seen in the pooled crystal structure data were deemed “successful”. Potential hydrogen bonds were assessed based on distances between the donor/acceptor heavy atoms of the test ligand pose and four key residues. Lengths measuring less than 3.3 Å were considered potential hydrogen bond interactions [52], [58]. Potential hydrophobic interactions were set to a distance threshold of 3.9 Å between carbon atoms [58]. Predicted interactions for each ligand were counted and a screen for the presence of hydrogen bond interactions with the key residues listed above was conducted to determine docking success.

### Ligand Binding Assay

ESA was introduced at various concentrations (0.001–10 µM) to solution containing PPARγ protein complexed with a fluorophore-bound compound (Fluormone™, Invitrogen). This mixture was allowed to incubate for 20 hours. The ability of the test compound, which here was ESA, to displace Fluormone™ was calculated as mean polarization, where a decrease in polarization corresponded to an increase in ligand binding activity as previously described [60].

### Transfection of RAW 264.7 cells

RAW 264.7 mouse macrophage precursor cells (ATCC, Manassas, VA) were grown in 24-well plates in DMEM high glucose medium (Invitrogen, Carlsbad, CA) containing 10% fetal bovine serum until 60–70% confluence. Transfected cells were treated with varying concentrations of ESA (0, 1, 5, and 10 µM; Sigma) or rosiglitazone (1 µM; Cayman Chemicals, Ann Arbor, MI) for 24 hours. Other details of the protocol were as previously described [60], [61]. Relative luciferase activity was calculated as a ratio between beginning and ending chemiluminescence values for a 10-second time period.

### Animal Procedures

The protocol for animal care and genotyping of the mice was described previously [8]. An ESA-supplemented diet was tested against a control (AIN-93G-based) diet in a dextran sodium sulfate (DSS)-induced IBD mouse model. Sixty mice were divided according to diet (ESA versus control), genotype (PPARγ flfl; MMTV-Cre-/PPARγ-floxed versus epithelial cell- and immune cell-specific PPARγ flfl; MMTV-Cre+/PPARγ-null), and DSS-challenge. Ten mice (5 for each genotype) from the control diet group and 9 mice (4 PPARγ-floxed and 5 PPARγ-null) from the ESA diet group were not given DSS-treated water as a control for the disease state. Drinking water with 2.5% DSS was administered to the test mice for a period of seven days. Body weights and disease activity index (DAI) values were recorded each day of the seven-day DSS treatment period. Procedures for assigning DAI values have been previously described [8]. Mice were euthanized on day seven of the DSS challenge by CO_2_ asphyxiation followed by secondary thoracotomy. Blood was withdrawn from the heart, after which spleen, mesenteric lymph nodes (MLNs), and colonic samples were examined for gross pathological lesions and isolated from each mouse. Organs were examined to assign scores based on size and macroscopic inflammatory lesions (0–3). Spleen and MLN were crushed to produce single-cell suspensions for flow cytometry, while colon samples were used for mRNA isolation and histological examination. This study was approved by the Virginia Tech Institutional Animal Care and Use Committee (IACUC) on May 15, 2008 under animal welfare assurance number A3208-01.

### Histopathology

Experimental design for histopathology was previously described [8], [20]. Epithelial erosion, mucosal thickness, and immune cell infiltration were each assessed and scored (0–4) for colon cross-sectional samples stained with hematoxylin and eosin from each mouse.

### Immunophenotyping

Whole blood and MLN cells were seeded onto 96-well plates and treated with fluorochrome-conjugated antibodies. Monocyte/macrophage subsets were assessed using anti-F4/80-PE-Cy5 (5 mg/mL, eBioscience) and anti-CD11b-Alexa Fluor 700 (2 mg/mL, eBioscience). The lymphocyte subset was assessed with anti-CD4-Alexa Fluor 700 (2 mg/mL; BD Pharmingen), anti-CD8-PerCp-Cy5.5 (2 mg/mL, eBioscience), anti-CD3 PE-Cy5 (2 mg/mL; BD Pharmingen), anti-FoxP3-PE (2 mg/mL, eBioscience), and anti-IL10-PE as previously described [62]. Flow results were computed with a BD LSR II flow cytometer and data analysis was performed with the FACS Diva software package (BD).

### Quantitative Real-Time RT-PCR

Total RNA was isolated from colonic tissue using procedures previously described [20]. PCR was performed on complementary DNA (cDNA) using Taq DNA polymerase (Invitrogen, Carlsbad, CA) and previously described methods and conditions [8], [20]. cDNA concentrations for genes of interest were examined by quantitative real-time PCR using an iCycler IQ System and the iQ SYBR green supermix (Bio-Rad). A standard curve was generated for each gene using methods previously described [20]. In addition, a melting curve analysis was performed for each product using previously described methods [20] in order to determine the number of products synthesized while excluding non-specific products and primer dimers. Real-time PCR was used to quantify the starting amount of nucleic acid of each unknown cDNA sample. Primer sequences, the length of the PCR product, and gene accession numbers have been outlined previously [20], [61]. Primers used for this study were the forward and reverse cohorts of VCAM-1, ICAM-1, IL-6, and β-actin [20].

### Statistical analysis

Data were analyzed as a completely randomized design with statistical significance assessed using the analysis of variance (ANOVA) method. The general linear model procedure of the Statistical Analysis Software (SAS) package (SAS Institute Inc., Cary, NC) was run for weight, DAI, flow cytometry data, and histopathology scores to determine variance across and significance between treatment groups. Statistical significance was assessed based on a probability value (*p*) less than or equal to 0.05. Significant models were further assessed using the Fisher's Protected Least Significant Difference multiple comparison method.

## Results

### Selection of structural model: Re-docking component

Structures with co-crystallized rosiglitazone (example given in Figure S1) were used for re-docking because rosiglitazone was the positive control in the experimental studies, it is a known PPARγ agonist, and the purpose of this docking feasibility test was to find compounds that mimic rosiglitazone-induced activation. The top scoring pose from each of the five 50-pose replicates was selected for further analysis. This selection method was applied for each of the five starting structures, giving a total of 25 poses for comparison.

The RMSD and free energy of binding were averaged for the five poses for each protein structure model (Table 1). Additionally, the population-based standard deviation and variance were calculated. The average pose RMSD values for three structures, 1FM6, 1ZGY, and 2PRG, were within 2.0 Å of the crystal structure position. Of these three, 1ZGY possessed the highest standard deviation and variance values, which suggested that some poses with low and high RMSD values should be present. Examination of the poses for all five structures revealed that the lowest RMSD value (0.99 Å) for all rosiglitazone poses was in the 1ZGY pose group as was the pose with the highest RMSD value (3.05 Å). Thus, we favored the 1ZGY structure for further docking studies because this structure enabled docking at the known rosiglitazone binding position as well as docking at other energetically favorable positions within the binding site, suggesting that it might accommodate ligands of diverse structure. To further confirm this selection, cross-docking with known ligands from other PDB structures was conducted.

**Table 1:** Average RMSD and free energy of binding (kcal/mol) for re-docking of rosiglitazone (N = 5).

		RMSD			kcal/mol		
PDB ID	Resolution (Å)	Mean	Standard Deviation	Variance	Mean	Standard Deviation	Variance
1FM6	2.1	1.76	0.561	0.314	−7.58	0.487	0.237
1ZGY	1.8	1.91	0.925	0.856	−7.19	0.247	0.061
2PRG	2.3	1.84	0.357	0.128	−7.66	0.228	0.052
3CS8	2.3	2.81	0.101	0.010	−6.63	0.184	0.034
3DZY	3.1	2.82	0.183	0.034	−7.06	0.133	0.018

### Selection of structural model: Cross-docking component

1ZGY, 1FM6, and 2PRG were included in the cross-docking testing as each showed successful re-docking and contained ligand-binding domains without missing loops or sequence segments. Structures 3CS8 and 3DZY were missing the H2′-H3 loop and did not result in accurate pose prediction for rosiglitazone. Rosiglitazone poses for 3CS8 and 3DZY occupied the portion of the binding cavity opening in which the H2′-H3 loop would normally sit (data not shown). This loop proved necessary for successful agonist docking given the poor success rate of re-docking in the absence of this region.

Vina was used for cross-docking instead of AD4 as the former was more time-efficient for the number of ligands used and the number of replicates to be carried out. It has also been reported that Vina better predicts poses for ligands with higher numbers of torsions [63], which was the case for some of the ligands used in cross-docking. Replicates were conducted with Vina for two reasons: to determine if replicates would be necessary in a larger-scale study, and to aid in the protein structure model selection process. Three replicate screens were run and each lowest-energy pose was analyzed (3 protein models×3 replicates×12 ligands = 108 lowest energy poses). Analysis of the cross-docking results included a comparison of RMSD values, free energy of binding, and number and identity of known interactions between each ligand and PPARγ based on the crystal structures of the complexes. Results from comparison of RMSD values and free energy of binding are listed in Table 2, with full ligand names listed in Table 3. To simplify the process of cross-docking of several ligands to multiple receptor structures, the initial crystal protein-ligand complexes were superimposed prior to docking. This practice allowed for RMSD values to be easily calculated between the docked ligand poses and crystal reference poses as the structures shared coordinate space.

**Table 2:** Average RMSD and free energy of binding from cross-docking for various ligands relative to each listed PDB ID (top row) (N = 3).

	1FM6			1ZGY			2PRG		
	RMSD (Å)								
PDB Ligand ID	Mean	SD^1^	Variance	Mean	SD	Variance	Mean	SD	Variance
243	2.82	0.199	0.040	2.82	0.014	0.000	2.60	0.040	0.002
570	3.19	0.000	0.000	3.08	0.007	0.000	3.13	0.050	0.003
4HD	1.81	0.365	0.134	1.40	0.018	0.000	2.19	0.236	0.056
9HO	1.73	0.184	0.034	1.85	0.270	0.073	1.70	0.162	0.026
DRH	2.74	0.030	0.001	1.55	0.209	0.044	2.17	0.251	0.063
DRJ	1.63	0.807	0.652	1.72	0.417	0.174	2.03	0.175	0.031
DRY	3.23	0.002	0.000	2.26	0.024	0.001	1.89	0.019	0.000
EHA	2.47	0.524	0.275	2.45	0.386	0.149	1.89	0.007	0.000
ET1	2.83	0.001	0.000	2.68	0.003	0.000	2.72	0.001	0.000
HXA	2.49	0.616	0.380	1.99	0.171	0.029	1.85	0.009	0.000
PTG-1	1.78	0.000	0.000	1.78	0.005	0.000	1.65	0.019	0.000
PTG-2	2.68	0.023	0.001	2.53	0.244	0.059	2.53	0.091	0.008

	Free energy of binding (kcal/mol)								
PDB Ligand ID	Mean	SD	Variance	Mean	SD	Variance	Mean	SD	Variance
243	−6.87	0.094	0.009	−6.57	0.047	0.002	−6.47	0.309	0.096
570	−10.43	0.047	0.002	−11.00	0.000	0.000	−10.50	0.082	0.007
4HD	−7.00	0.163	0.027	−7.53	0.170	0.029	−6.97	0.047	0.002
9HO	−6.40	0.082	0.007	−6.70	0.082	0.007	−6.37	0.094	0.009
DRH	−8.23	0.047	0.002	−8.83	0.125	0.016	−8.17	0.047	0.002
DRJ	−8.63	0.094	0.009	−8.80	0.294	0.087	−8.37	0.094	0.009
DRY	−10.03	0.047	0.002	−10.13	0.047	0.002	−10.37	0.047	0.002
EHA	−10.10	0.082	0.007	−10.10	0.082	0.007	−10.60	0.000	0.000
ET1	−8.10	0.000	0.000	−8.50	0.000	0.000	−8.50	0.000	0.000
HXA	−7.00	0.327	0.107	−7.90	0.082	0.007	−7.33	0.047	0.002
PTG-1	−7.00	0.000	0.000	−7.23	0.047	0.002	−7.20	0.082	0.007
PTG-2	−7.17	0.170	0.029	−7.10	0.082	0.007	−7.47	0.094	0.009

1 SD = Standard Deviation.

**Table 3:** Full names and structures for compounds listed by ligand ID in Table 2.

PDB Ligand ID	PDB ID	Reference	Ligand Name^1^
243	2VST	[46]	13-hydroxyoctadecadienoic acid(13-HODE)
570	1FM9	[37]	GI262570(Farglitazar)
4HD	2VV1	[46]	(4S,5E,7Z,10Z,13Z,16Z,19Z)-4-hydroxydocosa-5,7,10,13,16,19-hexaenoic acid(4-HDHA)
9HO	2VSR	[46]	9-hydroxyoctadecadienoic acid(9-HODE)
DRH	2I4P	[45]	(2S)-2-[4-[2-(1,3-benzoxazol-2-yl-heptyl-amino)ethyl]phenoxy]-2-methyl-butanoic acid((2S)-ureidofibrate-like derivative)
DRJ	2I4J	[45]	(2R)-2-[4-[2-(1,3-benzoxazol-2-yl-heptyl-amino)ethyl]phenoxy]-2-methyl-butanoic acid((2R)-ureidofibrate-like derivative)
DRY	2HWQ	[44]	[(1-{3-[(6-benzoyl-1-propyl-2-naphthyl)oxy]propyl}-1H-indol-5-yl)oxy]acetic acid(5-substituted indoleoxyacetic acid analogue)
EHA	2F4B	[43]	(5-{3-[(6-benzoyl-1-propyl-2-naphthyl)oxy]propoxy}-1H-indol-1-yl)acetic acid(Indol-1-yl acetic acid)
ET1	3ET3	[47]	3-[5-methoxy-1-(4-methoxyphenyl)sulfonyl-indol-3-yl] propanoic acid(indeglitazar)
HXA	2VV0	[46]	Docosa-4,7,10,13,16,19-hexaenoic acid
PTG	2ZK12ZK2	[48]	15-deoxy-delta(12,14)-prostaglandin J2 (PTG)

Ligand IDs from respective PDB files were used. Ligand structures can be found in Table S1.

1 Abbreviations for ligands mentioned in the text are in parentheses following the full name of the compound.

The results relative to each of the test structure models were not completely consistent across all the models. The lowest overall average RMSD was seen with 1ZGY for the (2S)-ureidofibrate-like derivative. This ligand did not dock as well into 1FM6 and 2PRG. A similar comparative docking pattern was seen for 4-HDHA. Only one ligand, PTG taken from PDB ID 2ZK1 (PTG-1), docked within the 2.0 Å threshold across the three structural models. It should be noted here that the PTG structure taken from PDB ID 2ZK2 possessed different charges than the same compound from 2ZK1. The difference in charge is most likely due to the difference in crystallization states. 2ZK2 had glutathione covalently bound to PTG-1 as part of crystallization, whereas 2ZK1 did not. The glutathione-PTG-1 compound would therefore have more atoms over which charges would be distributed.

The RMSD, standard deviation, and variance values for farglitazar, 9-HODE, indeglitazar, and PTG-1 showed the most consistency across the three proteins, with PTG-1 showing favorable average RMSD values and negligible variance for each protein structure. For PTG-1, this suggested the ligand docked similarly to all three protein structures. When the replicate poses for the four compounds were assessed visually, the deviations for the 9-HODE poses were due in large part to variation in the placement of the hydrophobic tail portion, the PTG-1 poses docked more similarly to 9-HODE than the PTG-1 reference structure, and the indeglitazar poses occupied the middle portion of the binding cavity rather than the rear activation site. The placement of the indeglitazar and PTG-1 poses appeared to be due to the shape of the binding cavity at the rear of the pocket, which was mentioned previously to be the issue with farglitazar. This hindrance was seen to a lesser degree with PTG-1 as there is sufficient space to allow interactions despite lack of exact congruence to the co-crystallized reference. Indeglitazar and farglitazar poses were consistently unsuccessful due to the binding cavity restriction, whereas PTG-1 occupied a fatty acid-like orientation given the similarity of this compound to the types of ligands that can appropriately fill the allotted molecular space.

All of the poses had negative calculated free energy of binding values given the ligand structures and charge environment of the binding cavity. These values were energetically feasible, but were not an indication of the most favorable conformation for ligands that did not agree with the reference structure geometry. Therefore, RMSD and free energy of binding measurements were not enough to determine successful cross-docking for PPARγ. A visual assessment of poses suggested rosiglitazone and fatty acid compounds dock the best into the selected models. As such, interactions from crystal structures containing these compounds were used to generate a list of favorable interactions that might indicate successful docking. The residues considered are listed in Table S4.

Inclusion of the interaction criteria improved the target structure model selection process. Based on the crystal structure interactions common to rosiglitazone and known fatty acid agonists, the number of possible interactions (Table S5) and instances of key residue hydrogen bonding (Table S6) were counted for all the poses. Both sets of data suggested that 1ZGY was the most appropriate model relative to 1FM6 and 2PRG for the purposes of this study. Poses docked into the 1ZGY model all showed at least one key interaction, whereas the other two models returned poses for some ligands that did not exhibit any known interactions. Additionally, fatty acid and fatty acid-derivatives returned the most favorable poses of all the cross-docking ligand types. If interaction analysis is included in the selection process, we see 1ZGY as the predominate candidate for the target structure model in a screen involving rosiglitazone-like and fatty acid compounds.

### Conjugated trienes showed association with PPARγ in silico

For the small-scale screen, a library of seventy-seven compounds was selected. These compounds included known active and inactive compounds, with alternate stereochemistry for some structures. This test set allowed for screening of active versus inactive, rosiglitazone-like versus non-TZDs, and molecularly simple versus complex compounds. The interaction data (Tables S7 and S8) reinforced the assumption that the selected target structure model could accommodate rosiglitazone-like and fatty acid compounds. The cross-docking ligands included in the screen docked similarly to what was seen with the cross-docking test. Most of the rosiglitazone-like compounds studied by Markt et al. [55] showed successful docking. These compounds were Chemical Abstracts Service (CAS)# 264908-13-6, CAS# 651724-09-3, CAS# 853652-40-1, BRL48482, BVT13, CLX-M1, KRP297, and NNC61-4424 (Table S1). Isomers of these compounds with differences in stereochemistry were used as well. Some of these structures did not dock as well, which was expected given it has been suggested from crystal structure studies that chirality can affect agonist activity [48]. We also saw lack of favorable docking for bulkier compounds, which contain multiple ring and aromatic components, and compounds with multiple hydroxyl groups. These ligands included phenolic extracts taken from *Glycyrrhiza glabra* roots isolated by Kuroda et al. [54], α-santonin-derived compounds identified by Tanrikulu et al. [53], and flavonoids screened by Salam et al. [52] (Table S1). The compounds from Kuroda et al. [54] and Tanrikulu et al. [53] compounds were numbered according to extraction fraction and deviation from the original α-santonin scaffold, respectively. The Kuroda et al. subset included compounds that induced low level activation. The Tanrikulu et al. subset contained one highly active compound (Tanrikulu_1), one moderately active compound (Tanrikulu_2), and six inactive compounds (Tanrikulu_3 through Tanrikulu_8). The selected Salam et al. compounds were apigenin, biochanin-A, chrysin, dihydroquercetin, genistein, hesperidin, psi(ψ)-baptigenin, and vitexin. The unsuccessful docking of known active compounds in these groups indicated the receptor structure was not appropriate for docking of these molecule types.

All of the conjugated trienes docked successfully but with similar geometry and energy scores, so a more detailed test for these compounds was conducted to see if a predominant ligand could be identified. AD4 was used to dock jacaric, catalpic, calendic, eleostearic, and punicic acids into the selected structural model, 1ZGY. Three iterations of 50 poses each were run and the lowest energy pose for each run for each fatty acid was selected and compared (15 lowest energy poses). The numbers of potential hydrogen bonds and hydrophobic interactions for each pose were calculated (Table S8). The lowest energy pose with the most potential hydrogen bond interactions was selected for each triene and used for analysis. As there are no crystal structures available with any of these compounds co-crystallized, interactions from PDB structures with fatty acids bound were used to generate an interaction reference list (Table S3). The four key residues that formed hydrogen bonds with rosiglitazone also formed hydrogen bonds with these fatty acids. Therefore, poses that possessed these interactions were deemed successful agonists. Unsuccessful poses were those lacking the agonist interactions and poses with the reactive polar group pointed away from the activation site.

**Figure 1:**
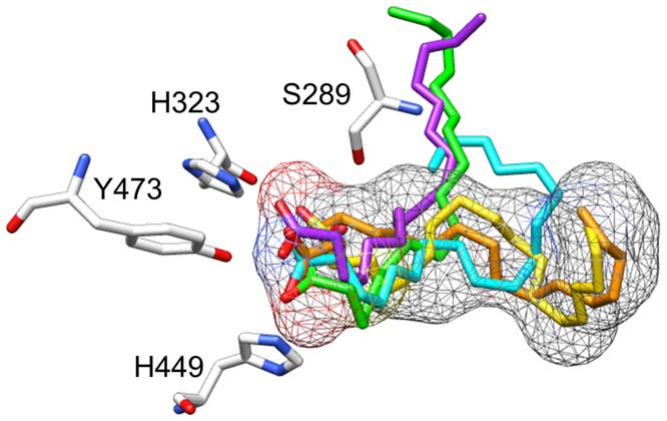
Predicted docked conformations for α-eleostearic (purple), punicic (cyan), calendic (orange), jacaric (green), and catalpic (gold) acids relative to the rosiglitazone-occupied portion of the binding cavity (mesh surface) in the rigid PPARγ structure model. Key residues with which hydrogen bonding occurs are labeled. Atom-specific coloring: red = oxygen; gray = carbon; blue =  nitrogen. Table 4 contains distance measurements for each docked pose.

All the conjugated trienes showed favorable docked poses and exhibited interactions with residues associated with PPARγ activation (Table 4). The triene poses occupied a space similar to that seen with rosiglitazone (Figure 1), and exhibited interactions with key residues. Of all the replicate poses for triene docking, the ESA replicates consistently exhibited the most negative free energy of binding (Table S9). Hydrogen bond interactions with only two of the four key residues were seen; however, it is not clear if interactions with all four residues are absolutely necessary for activation, or if a reduced number of interactions can still induce activation. It is feasible that a reduced number of specific interactions may contribute to the specificity seen with ligand-induced co-activator recruitment for PPARs. A comparison of distance measurements for the interactions showed two Y473-involved interactions for ESA, punicic acid, and jacaric acid. Given the distance measurements, it was proposed that the acid head group straddles Y473, with one oxygen atom closer to one histidine side chain than the other. This was confirmed when the poses were visually assessed. The number of hydrophobic interactions was more consistent for the ESA poses compared to punicic and jacaric acids. As previously mentioned, it is known that punicic acid binds to PPARγ and modulates its activity, while ESA possesses greater antioxidant effects. Given the combination of what was known experimentally about the compounds and the predicted free energy of binding and interactions, ESA was selected as a candidate for validation using a ligand-binding assay and further experimental testing *in vivo*.

**Table 4:** Distance measurements (in Angstroms [Å]) for docked conjugated triene poses displayed in Figure 1.

Ligand	Color	Residue	Distance (Å)	kcal/mol
eleostearic acid	purple	H323.NE2	3.16	−5.6
		Y473.OH	3.01	
		Y473.OH	3.27	
punicic acid	cyan	H449.NE2	2.84	−4.28
		Y473.OH	3.03	
		Y473.OH	3.07	
calendic acid	orange	H449.NE2	2.81	−4.47
		Y473.OH	3.10	
catalpic acid	gold	S289.OG	3.05	−4.48
		H323.NE2	3.03	
		Y473.OH	3.26	
jacaric acid	green	H449.NE2	2.84	−4.5
		Y473.OH	3.16	
		Y473.OH	3.10	
rosiglitazone	gray mesh	S289.OG	3.02	N/A
		H323.NE2	2.83	
		H449.NE2	3.02	
		Y473.OH	2.85	

Distances were measured between carboxylic oxygen atoms of fatty acids and listed atoms for each residue. Free energy of binding is measured in kilocalories per mole of ligand (kcal/mol). No value is listed for rosiglitazone as this refers to the crystal conformation (denoted “N/A”) Residues are labeled as the amino acid designation plus the atom name (e.g., S289.OG refers to the oxygen atom in the gamma position on serine 289).

### ESA bound to and modulated PPARγ in vitro

**Figure 2:**
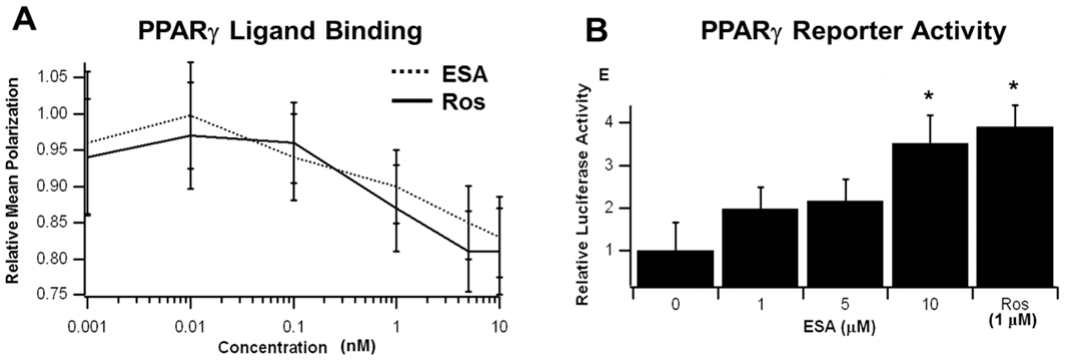
Ligand-binding (A) and reporter assay (B) results for ESA bound to PPARγ with rosiglitazone (Ros) as a positive control. (A) Ligand binding was assessed as a measure of mean polarization for the displaced Fluormone™ molecule versus increasing concentrations of either ligand. (B) Reporter activity was measured as relative luciferase activity for various concentrations of ESA versus 1 µM Ros. Error bars represent standard deviation, while asterisks (*) indicate significance (*p*≤0.05) between the data sets.

The results of our molecular docking efforts and various published studies [24], [26]–[31], [33], [34] indicated that conjugated trienes, specifically ESA, may bind to and modulate PPARγ activity. Ligand-binding and reporter activity assays were conducted to test this assumption. A cell-free ligand-binding assay was implemented to determine if ESA associated with PPARγ *in vitro* and possessed a similar depolarization pattern to rosiglitazone. The results suggested the depolarization pattern for ESA was similar to that seen with the rosiglitazone positive control with no significant difference between the two curves (Figure 2A).

An assessment of PPARγ activity modulation was conducted using RAW 264.7 cells and varying ESA concentrations (0–10 µM). Relative luciferase activity was measured to determine ligand-induced activation. The reporter assay suggested ESA does modulate PPARγ activity, but at a concentration 10-fold higher than the rosiglitazone control (Figure 2B), suggesting that there may be a difference in either potency or uptake by the cells between both compounds.

### ESA ameliorated clinical signs of IBD

**Figure 3:**
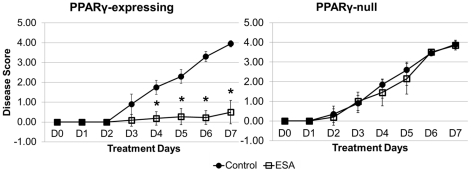
Effect of ESA on disease activity scores for PPARγ-expressing (A) and PPARγ-null (B) mice with experimental IBD. PPARγ-null refers to lack of functional PPARγ product in colon epithelial and immune cells only. Data points represent averaged disease scores for each group with error bars representing standard deviation. Asterisk (*) indicates significance (*p*≤0.05).

Under our DSS-induced IBD model, ESA significantly ameliorated IBD in mice with the wild phenotype (i.e., PPARγ-floxed). This observation was based on the significant difference between DAI for the last four days of the seven-day challenge (Figure 3). IBD-related disease phenotypes were milder in the ESA-fed PPARγ-expressing group of mice compared to the ESA-fed cell-specific PPARγ-null mice. The control groups (no ESA) for both genotypes showed no improvement in IBD phenotypes over the seven-day time course. Therefore, ESA was effective in ameliorating disease-associated phenotypes in mice with DSS colitis through a PPARγ-dependent mechanism.

### Immunophenotypes for harvested tissues

**Figure 4:**
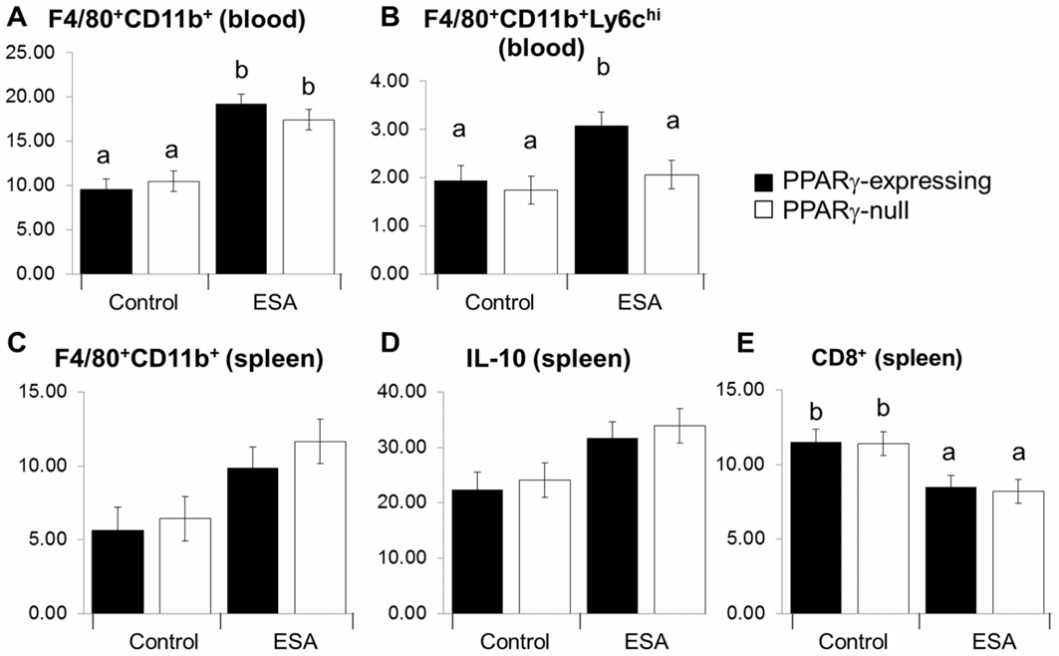
Effect of ESA on immune cell subsets of PPARγ-expression and PPARγ-null mice with experimental IBD. Tissues examined included blood (A and D) and spleen (B, C, and E). Values represent least square means for percentage of gated cells with error bars to indicate standard error. Letters indicate significance (*p*≤0.05) where a shared letter indicates groups which are not statistically significantly different.

Changes in immune cell subsets due to DSS-induced colitis were assessed in the harvested tissues to investigate the modulation of inflammation by ESA (Figure 4). Flow cytometry was used to characterize the phenotype of macrophages and T cell subsets. DSS augmented the percentages of monocytes or macrophages in the blood and spleen (Figure 4A and 4C). A significant increase in blood monocytes was found in ESA-treated mice. The PPARγ-expressing mice on the ESA diet exhibited a higher percentage of monocytes expressing lymphocyte antigen 6 complex-high (Ly6C^hi^), which was not seen in the PPARγ-null group (Figure 4B) indicating a PPARγ dependency of this effect. Higher levels of IL-10 were observed in the spleen of the ESA-fed mice for both genotypes although these numerical differences were not statistically significant between the two diets for the PPARγ-expressing genotype (Figure 4D). Lastly, we found a numerical decrease in CD8^+^ T-cells in the ESA diet group (Figure 4E), where the change was PPARγ-independent.

### Histological trends mimicked clinical activity

**Figure 5:**
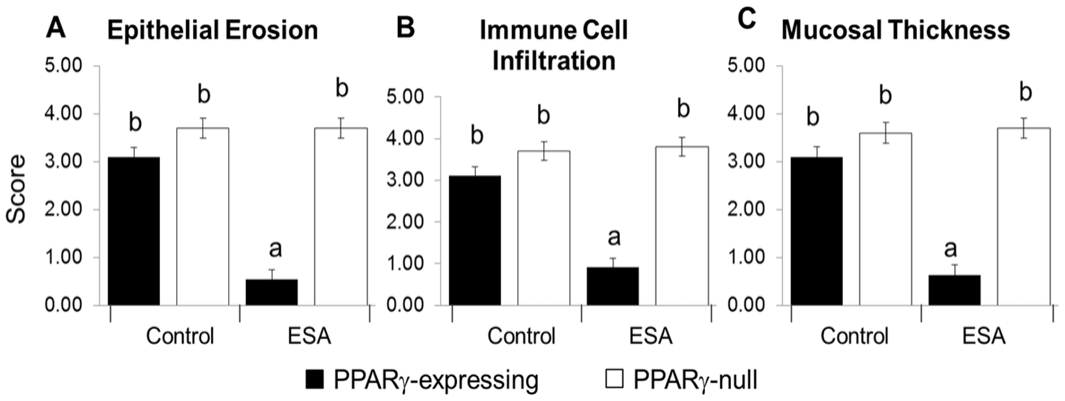
Effect of ESA on histopathological lesions in colons from PPARγ-expressing and PPARγ-null mice with experimental IBD. Epithelial erosion (Erosion) (A), immune cell infiltration (Infiltration) (B), and mucosal thickness (Thickness) (C) were assessed and averaged for all the DSS-treated group of samples. Data are presented as mean score with error bars to indicate standard deviation. Letters indicate significance (*p*≤0.05) where a shared letter indicates groups which are not statistically significantly different.

There was a significant decrease in epithelial erosion (Figure 5A), mucosal thickness (Figure 5B), and immune cell infiltration (Figure 5C) in the ESA-fed PPARγ-expressing mice but not in ESA-fed PPARγ-null mice. This suggested amelioration of experimental IBD phenotypes by ESA is PPARγ-dependent. This agreed with the DAI data and further indicated an ESA-associated PPARγ-dependent improvement in IBD phenotypes.

### Gene expression suggested PPARγ-dependent and -independent mechanisms

**Figure 6:**
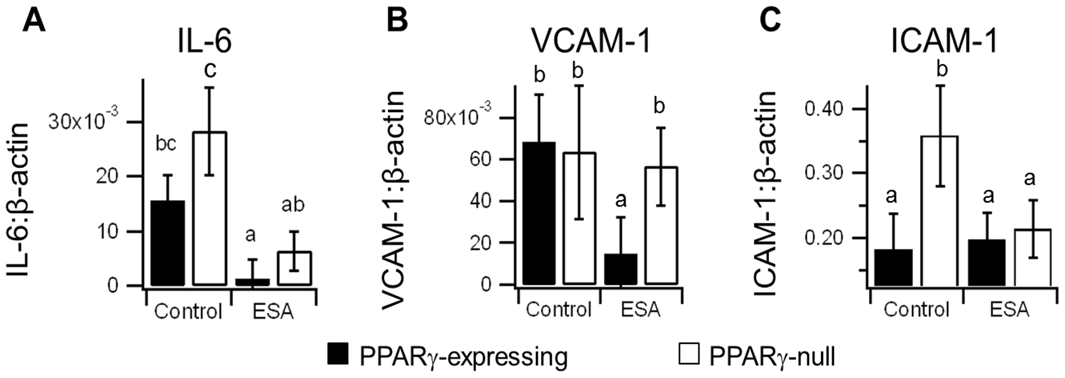
Effect of ESA on colonic concentrations of IL-6 (A), VCAM-1 (B), and ICAM-1 (C) in PPARγ-expressing and PPARγ-null mice with experimental IBD. The mean ratio of expression for each protein relative to constitutively expressed β-actin is shown with error bars to indicate standard deviation. Letters indicate significance (*p*≤0.05) where a shared letter indicates groups which are not statistically significantly different.

There was a marked decrease in IL-6 and VCAM-1 mRNA expression between the control- and ESA-fed PPARγ-expressing groups (Figure 6A and 6B). The IL-6 decrease appeared to be PPARγ-independent, while the VCAM-1 decrease was PPARγ-dependent. We also found a decrease in ICAM-1 expression between the control and ESA diet groups, but this decrease also occurred in the PPARγ-null mice suggesting ESA can induce ICAM-1 regulation in a PPARγ-independent manner (Figure 6C).

## Discussion

The VS model protein structure and parameters used in this study allowed for prediction of docking conformations for rosiglitazone-like and fatty acid compounds. The re-docking results for rosiglitazone, cross-docking results for PTG-1 and 9-HODE, and the conjugated triene docking all suggested 1ZGY is appropriate for screening fatty acids and TZD-like compounds. Potential for docking of fatty acid derivative partial agonists, like (2S)-ureidofibrate-like derivative, was also seen, but not fully assessed for this study as full agonism was the binding type of interest. Thus, we have successfully established a VS parameter set appropriate for a large-scale PPARγ full agonist search amongst fatty acids and fatty acid derivatives.

Information regarding interactions known to occur with PPARγ agonists is a suitable means to identify docking success. However, the success rate may be improved by incorporating even more criteria. Such criteria include a more extensive list of key interactions and/or establishment of distinct lists to specify interactions characteristic of each ligand category (e.g., full agonist, partial agonist, and antagonist). Based on the number of interactions and presence of interactions with key residues, we were able to determine which ligand types do and do not fit our selected target structure model. Combining this with RMSD data allowed us to see which types of ligands dock away from the binding cavity given the molecular environment of the selected target structure model. This information regarding ligands that would be excluded in a screen for compounds that interact similarly to what is seen with rosiglitazone can be used to identify one or more additional target structure models to incorporate into a large-scale screen. RMSD data, however, would not be available from a screen of unknowns, and conclusions would therefore have to be drawn from the interaction and free energy data.

Due to the high degree of precision observed with the cross-docking ligands, it was determined that a single pose for each ligand would be sufficient for the initial analysis step in a large-scale screen. Replicates were necessary for the pre-screening analysis in which parameters and structure models were tested for predictability. Replicates are useful in docking studies to ensure any conclusions are based on consistent interactions. However, running replicates for a library numbering in the thousands is computationally time-consuming and less than practical given replicate poses may possess geometry that is exactly or close to the same. Rather than run replicates on the entire library of compounds, it would be feasible to run more detailed docking with compounds selected as successful binders of interest with the potential for experimental verification.

We observed a complementary relationship between the experimental ESA-IBD study and the computational screening results. In a recent review, we mentioned previous studies in which dual- or pan-agonistic effects have been associated with conjugated trienes [64]. This information, coupled with other published studies regarding synthetic agonists and inactive compounds, provided a means to develop and test computational methods for identifying natural agonists. Our docking analysis suggested ESA possessed a more favorable binding energy compared to the other conjugated trienes. Though comparative relationships have not been established between ESA and all the tested trienes, we do know that ESA possesses greater antioxidant effects than punicic acid in mice [65]. It is plausible that the differences in efficacy between the compounds is interaction-related, which may result in conformational changes that attenuate co-activator recruitment and subsequent transcriptional regulation. The interaction aspect may have been picked up by our study, but the dynamic significance was not. This second aspect would require further computational testing to see if differences in protein stability and conformation can be detected between the protein-ligand complexes.

The ligand binding and reporter assays verified that ESA binds to and modulates PPARγ. Our docking study suggested fewer interactions occurred in the PPARγ-ESA complex compared to PPARγ-rosiglitazone. It is possible that the absence of interactions with S289 and H449 could result in a different level of ligand-induced activity attenuation or the interactions with H323 and Y473 may be more important for fatty acid-induced agonism. Given the different levels of agonism, which is ligand-dependent, it is plausible that the specificity toward anti-inflammatory mechanisms observed as PPARγ-dependent in the pre-clinical trial were influenced by some difference in agonism specific to ESA. This notion is further supported by the absence of rosiglitazone-associated phenotypes seen in studies published by other groups [65], [66]. Both the Shah et al. and Ramakers et al. studies involved testing rosiglitazone against DSS-induced colitis in mice [65], [66]. Ramakers et al. showed weight gain in mice treated with rosiglitazone prior to DSS challenge, followed by significantly greater weight loss compared to the control after DSS challenge [66]. Increases in the severity of colitis-specific colon phenotypes were also seen, but with a decrease in inflammation [66]. The Shah et al. study indicated a PPARγ-dependent rosiglitazone-induced decrease in macrophage recruitment, but showed no other significant changes to the levels of other cytokines [65].

We have shown that the immune modulatory actions of ESA may be both PPARγ-dependent and PPARγ-independent in mice with experimental IBD, although its effects on disease activity and colonic lesions are dependent on expression of PPARγ by immune and epithelial cells. It is known that PPARγ is highly expressed in immune cells, intestinal epithelial cells (IECs), and adipocytes, with lower expression levels throughout various tissues of the body. Recently, our group published work in which the severity of IBD was tested in a mouse model for IEC-specific PPARγ deletion in a C57BL/6 background [67]. It was determined that the absence of PPARγ from IECs resulted in significantly worse disease scores, greater loss of body weight, and increased inflammation in the colon, spleen, and MLN compared to mice expressing PPARγ [67]. Further, it was concluded that the presence of PPARγ in IEC contributes to anti-inflammatory effects, regulation of immune cell distribution, and gene expression regulation necessary to counteract IBD symptoms [67].

Additionally, there are studies in which PPARγ expression and the effect of ESA on disease pathogenesis have been evaluated in breast cancer cell lines [27], [68], pre-adipocytes [69], and colon cancer cell lines [70]. In all cases the fatty acid was capable of significantly ameliorating the disease via PPARγ-dependent responses such as induced apoptosis of cancer cells [27], [68], [70] and reduced lipid storage during differentiation [69]. Other conjugated trienes, such as punicic acid and catalpic acid [23], [24] have shown reduced inflammation responses in cancer, cardiovascular disease [71], and obesity [23], [24], [71]. All of these studies are strong examples of how PPARγ mediates inflammatory, metabolic, proliferation, signal transduction, and cellular motility processes [67] in various cell types.

It is possible that the presence of other nuclear receptors in the cells play a role in ESA-mediated effects. PPARδ in the colon may play a role in ESA-mediated IBD amelioration given the possibility of dual-agonist and pan-agonist modulation seen with PPARs, and the ability of all three PPARs to accommodate fatty acids. Further computational and experimental tests would be necessary to determine whether ESA mediates both PPARγ and PPARδ transcriptional regulation, which has been previously described for CLA [72]. The anti-inflammatory responses induced by ESA, which appeared to be PPARγ-independent, might also be attributed to other unforeseen targets in the system. For instance, we previously described the potential of PPARγ agonists to bind to lanthionine synthetase component C-like protein 2 (LANCL2) [60]. Such an association is one proposed molecular mechanism of regulating disease-related inflammatory effects in a PPARγ-independent manner.

Beyond what is seen in IBD, it has been shown that ESA binds to and activates estrogen receptors in breast cancer cell lines [73]. It is also known that hepatocyte nuclear factor-4α (HNF4α), which is essential for maintaining lipid homeostasis via gene regulation and regulating hepatocyte differentiation, is activated by fatty acids [74]. It has been suggested that PPARα ligands can interfere with HNF4α activity [75], but the mechanism by which this occurs is not fully understood. As conjugated trienes like punicic acid activate PPARα in adipocytes [24], and PPARα and fatty acids are present in liver tissue also, it seems feasible that conjugated trienes could come in contact with and bind HNF4α as well. To our knowledge such a study involving HNF4α and ESA or any other conjugated trienes has not been conducted.

**Figure 7:**
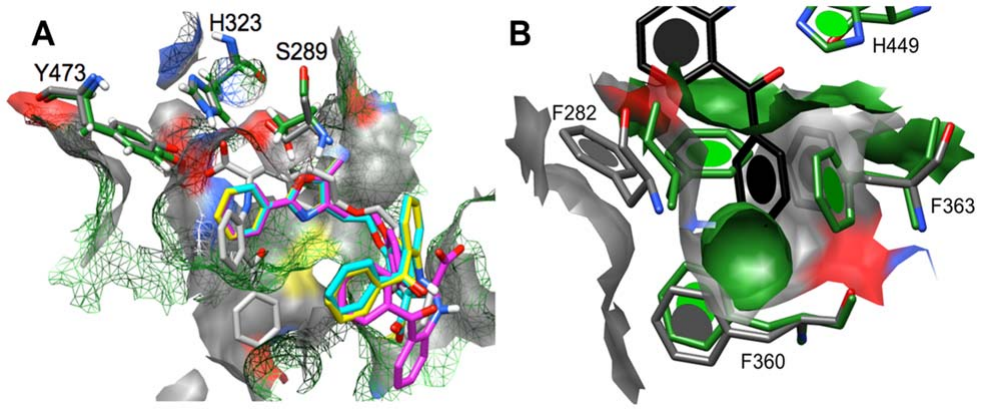
Visual assessments of molecular surface differences that result in unsuccessful docking of specific ligand types to the selected PPARγ structure model. Farglitazar is represented in both panels with atom-specific coloring. (A) 1ZGY and 1FM9 surface representations are green mesh and solid gray, respectively. The three poses predicted for farglitazar relative to 1ZGY are shown in magenta, cyan, and yellow. (B) Side chain rotamers for F282 and F363 are responsible for the differences in cavity surface at the rear of the cavity. Surface colors for 1ZGY and 1FM9 are the same as in (A). Atom-specific coloring: gray/black = carbon, blue = nitrogen, red = oxygen, white = hydrogen, and yellow = sulfur.

The ability of the binding cavity to accommodate many different ligand types represents a major technical obstacle when performing computational docking into PPARγ as a therapeutic target. The issue stems from the dynamic nature of the binding cavity and changes in protein conformation necessary to accommodate different agonists. This dynamic nature is not possible with rigid macromolecule docking techniques, and incorporation of flexibility can be difficult given the number of residues that can possess variable positions and the number of possible rotamers for each residue. The rigidity of crystal structures combined with the variability of residue side chain positions proved an issue for docking non-native ligands to the selected structure model. For example, the docked poses for farglitazar across the three protein structure models examined in the cross-docking step reflected a lack of appropriate molecular volume at the rear of the binding pocket to accommodate the benzyl ketone group on the ligand (Figure 7A). When the three structure models were compared to the 1FM9 crystal structure in which farglitazar was co-crystallized, the space necessary to accommodate the benzyl ketone group of farglitazar was missing given the differences in the side chain positions for Phe282 and Phe363 (Figure 7B). These residues do not pose an issue for rosiglitazone docking, but occupied the portion of the cavity in which farglitazar should have docked, which prevented successful cross-docking of this compound to the selected structure models. As such, selection of a single model to appropriately accommodate a narrow range of ligands and selection of several models to use with a diverse ligand library are two avenues toward identifying PPARγ agonists *in silico*. The first technique is used widely, but the second is not as common due to the amount of time necessary to properly identify target structure models. Given the molecular exclusion of the more hydrophobic compounds in our small-scale screen, the second technique would be ideal for dealing with a diverse library, such as the one we have constructed. Therefore, further testing with additional protein structure models capable of accommodating bulkier and more hydrophobic compounds would be necessary.

An additional technique for improving predictability is molecular dynamics simulation and analysis, which is also extremely time consuming and can prove problematic since parameters for ligands must be developed. Conformational sampling of the PPARγ binding cavity via MD is one means of gleaning useful information in a relatively short amount of time. This technique would provide information about predominant conformations adopted by PPARγ that would aid in the selection of multiple target structure models for docking, and can be easily verified by the large number of available crystal structures.

PPARγ has proven a difficult protein to explore as a drug target given dynamic and specificity issues. The large binding cavity and ability of the protein to accommodate a wide range of compounds presents an issue for rigid docking screening. The ability of the protein to bind compounds of different compound families requires a degree of ligand diversity that is often not employed in conventional VS studies. As a means to improve our method, we are currently testing additional PPARγ crystal structures as docking targets. As a consequence of this study, we have established a need for at least one additional target structure model that can accommodate bulker compounds. An analysis of MD simulations for unbound active, bound active, and unbound inactive forms of PPARγ are ongoing. These simulations, combined with further analysis of available crystal structure models, will allow us to develop additional target structure models. Incorporating conformational variability by screening against multiple protein conformations of the same protein should improve our screening process. We propose matching ligand and protein pharmacophores prior to screening to reduce the incidence of screening ligands against a protein structure into which the ligands cannot fit or where the charge environment is inappropriate.

The diversity of our compound database is being expanded as well, and will include an extensive list of known PPARγ agonists, decoy compounds that mimic known agonist structure but are inactive toward PPARγ, drugs currently available for treatment of other diseases, and extracts tested experimentally for PPARγ modulation. Such a library would improve enrichment, which is part of the separation of binders from non-binders. Further, inclusion of a weighting system based on the occurrence of known interactions would improve the separation process. With a diverse library in which available therapeutics are included, it may be possible to identify lesser known drug interactions with PPARγ linked to side effects seen with patients taking medications for cancer and neurological diseases. Given the success of our current study and the pending improvements to our method for testing of diverse ligand types, we are making progress toward an extensive and highly effective means to computationally identify feasible PPARγ-targeted drug candidates. Ideally, the established methods could be applied to the other PPARs, other nuclear hormone receptors, and alternate protein family targets where similar considerations must be made.

This study exemplifies how experimental methods can be used to complement and verify computational predictions. We have demonstrated that it is possible to predict ligand association given information known about the binding cavity of the target. We have also established a means to reduce the need for researcher intervention in assessing successful binding by incorporating a search for key interactions. More specifically, we have successfully established a protocol for screening fatty acid compounds against PPARγ for agonism, and were able to predict that ESA and other conjugated trienes would bind to and activate PPARγ using molecular docking. These predictions have been verified through *in vitro* assays both here and in our previous work [24], [34]. *In vivo* efficacy was assessed as well to determine if disease-associated benefits could be seen given the activation of PPARγ by ESA. In this regard, ESA did induce both PPARγ-dependent and -independent responses that ameliorated disease activity and intestinal lesions in IBD. The scope of this work implies the techniques described here can aid in streamlining drug discovery and development techniques as the technology develops.

## Supporting Information

Figure S1 Colored ribbon representation of PPARγ showing three layers of helical “sandwich”, and co-crystallized rosiglitazone (PDB ID 1FM6 [40]). Helices for each layer are colored, with helix H12, which sits at the rear of the binding cavity (AF-2 region), colored in red. Rosiglitazone is colored in green, with oxygen, nitrogen, and sulfur atoms colored red, blue, and yellow, respectively. The insert (upper right) shows a close-up view of the molecular surface of the binding cavity. The thiazolidinedione head group of rosiglitazone sits at the rear of the binding cavity where it can interact with S289, H323, H449, and Y473 in order to change the conformation of the AF-2 region and activate the protein.
(TIF)

Table S1 List of ligands used for virtual screening.
(DOC)

Table S2 List of atoms for key residues common to selected rosiglitazone crystal structures used to assess potential interactions between docked poses and the protein structure model.
(DOC)

Table S3 List of atoms for key residues common to selected fatty acid-bound crystal structures used to assess potential interactions between docked poses and the protein structure model.
(DOC)

Table S4 List of atoms for key residues common to rosiglitazone- and fatty acid-containing PDB structures used to assess potential interactions between docked poses and the protein structure model.
(DOC)

Table S5 Predicted hydrophobic and hydrogen bond interactions for ligands in cross-docking test set relative to a reference list of interactions common to rosiglitazone and selected fatty acids. Poses were taken from docking of each ligand into each of the three listed PPARγ PDB files (top row). Ligand IDs refer to compounds listed in Table 3.
(DOC)

Table S6 Presence or absence of potential hydrogen bond interactions between indicated residues of selected protein structure models and replicate poses of ligands listed by ID. A single “x” indicates one potential interaction for the listed residue was found for the specified ligand, whereas more than one “x” indicates more than one interaction (e.g., “xx” indicates two interactions found). (N = 3)
(DOC)

Table S7 Predicted hydrophobic and hydrogen bond interactions for ligands in small-scale screening test set relative to a reference list of interactions common to rosiglitazone and selected fatty acids (Table S4). Poses were taken from docking of each ligand into each of the three listed PPARγ PDB files (top row). Predicted free energy of binding is listed as kcal/mol.
(DOC)

Table S8 Presence or absence of potential hydrogen bond interactions between indicated residues of selected protein structure models (top row) and ligand poses. A single “x” indicates one potential interaction for the listed residue was found for the specified ligand, whereas more than one “x” indicates more than one interaction (e.g., “xx” indicates two interactions found).
(DOC)

Table S9 Predicted free energy of binding and interaction counts for conjugated trienes. Docking was performed using AD4 with three top-binding replicates for each ligand (150 total conformations). The highest energy conformation with the highest number of hydrogen bonds was used for analysis in Table 4.
(DOC)

Formulas S1 (DOC)
