# Supplementary figures and images for: Dietary α-Eleostearic Acid Ameliorates Experimental Inflammatory Bowel Disease in Mice by Activating Peroxisome Proliferator-Activated Receptor-γ
Source: PLoS One. 2011 Aug 31;6(8):e24031. doi: 10.1371/journal.pone.0024031 (PMC3164124; doi:10.1371/journal.pone.0024031)

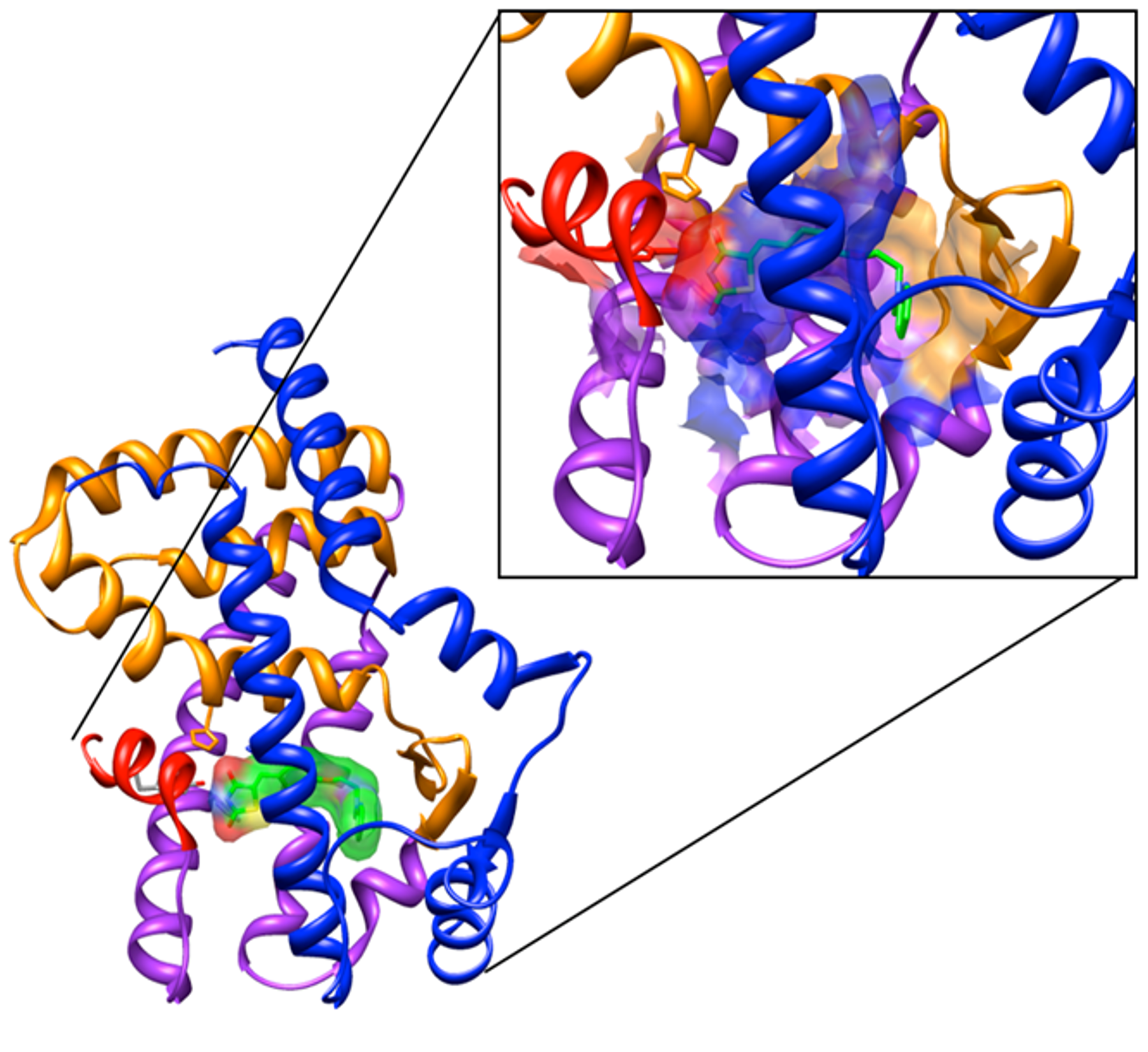

Supplement: Figure S1 — Colored ribbon representation of PPARγ showing three layers of helical “sandwich”, and co-crystallized rosiglitazone (PDB ID 1FM6 [40]). Helices for each layer are colored, with helix H12, which sits at the rear of the binding cavity (AF-2 region), colored in red. Rosiglitazone is colored in green, with oxygen, nitrogen, and sulfur atoms colored red, blue, and yellow, respectively. The insert (upper right) shows a close-up view of the molecular surface of the binding cavity. The thiazolidinedione head group of rosiglitazone sits at the rear of the binding cavity where it can interact with S289, H323, H449, and Y473 in order to change the conformation of the AF-2 region and activate the protein. (TIF) [file pone.0024031.s001.tif]
