# Supplementary material for: Dietary α-Eleostearic Acid Ameliorates Experimental Inflammatory Bowel Disease in Mice by Activating Peroxisome Proliferator-Activated Receptor-γ
Source: PLoS One. 2011 Aug 31;6(8):e24031. doi: 10.1371/journal.pone.0024031 (PMC3164124; doi:10.1371/journal.pone.0024031)
Supplement: Table S1 — List of ligands used for virtual screening. (DOC) [file pone.0024031.s002.doc]

**Table S1** List of ligands used for virtual screening.

| Ligand | Activity | Reference | Structure |
| --- | --- | --- | --- |
| GI262570 (Farglitazar) | active |  | 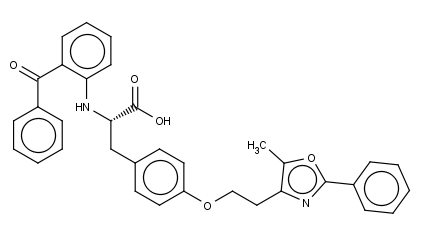 |
| 9-hydroxyoctadecadienoic acid | active |  | 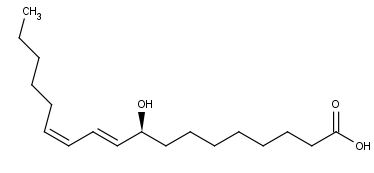 |
| 13-hydroxyoctadecadienoic acid | active |  |  |
| (4S,5E,7Z,10Z,13Z,16Z,19Z)-4-hydroxydocosa-5,7,10,13,16,19-hexaenoic acid | active |  | 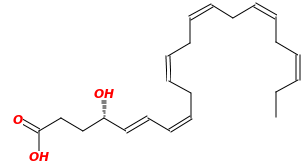 |
| Docosa-4,7,10,13,16,19-hexaenoic acid | active |  | 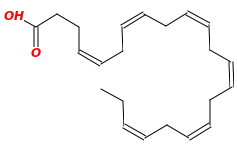 |
| 15-deoxy-delta(12,14)-prostaglandin J2 | active |  | 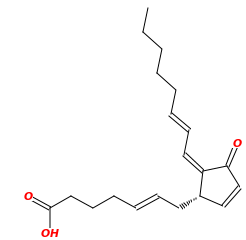 |
| (5-{3-[(6-benzoyl-1-propyl-2-naphthyl)oxy]propoxy}-1H-indol-1-yl)acetic acid | active |  |  |
| [(1-{3-[(6-benzoyl-1-propyl-2-naphthyl)oxy]propyl}-1H-indol-5-yl)oxy]acetic acid | active |  |  |
| (2R)-2-(4-{2-[1,3-benzoxazol-2-yl(heptyl)amino]ethyl}phenoxy)-2-methylbutanoic acid | active |  |  |
| (2S)-2-(4-{2-[1,3-benzoxazol-2-yl(heptyl)amino]ethyl}phenoxy)-2-methylbutanoic acid | active (partial agonist) |  |  |
| 3-[5-methoxy-1-(4-methoxyphenyl)sulfonyl-indol-3-yl]propanoic acid | active |  | 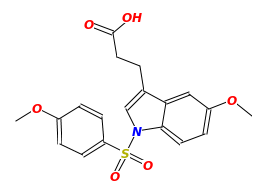 |
| (9Z,11E)-octadeca-9,11-dienoic acid (CLA) | active |  | 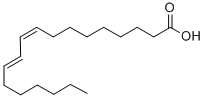 |
| Calendic Acid | active |  |  |
| Catalpic Acid | active |  |  |
| Jacaric Acid | active |  |  |
| Kuroda_No10 | inactive |  | 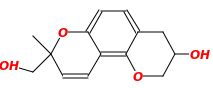 |
| Kuroda_No15 | inactive |  | 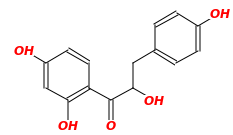 |
| Kuroda_No16 | active |  | 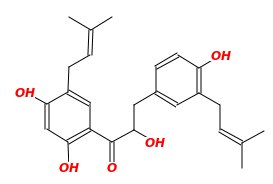 |
| Kuroda_No2 | active |  | 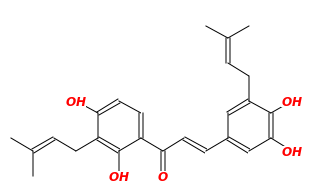 |
| Kuroda_No34 | active |  | 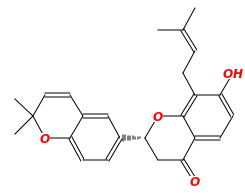 |
| Kuroda_No38 | active |  | 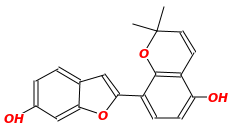 |
| Kuroda_No39 | active |  | 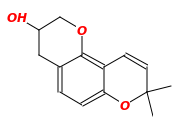 |
| Kuroda_No3 | active |  | 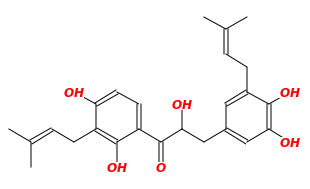 |
| Kuroda_No5 | active |  | 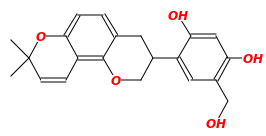 |
| Kuroda_No6 | active |  | 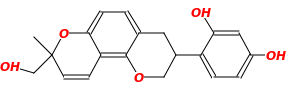 |
| Markt_264908-13-6_1 | active |  | 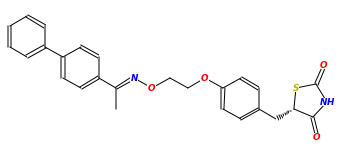 |
| Markt_264908-13-6_2 | active |  | 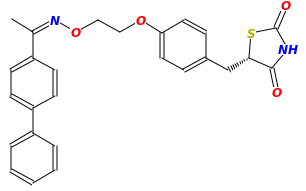 |
| Markt_264908-13-6_3 | active |  | 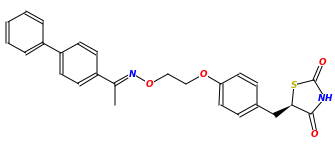 |
| Markt_264908-13-6_4 | active |  | 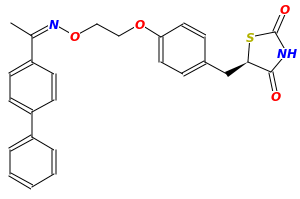 |
| Markt_651724-09-3_1 | active |  | 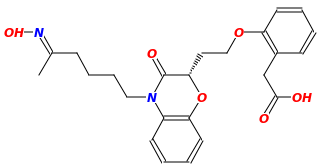 |
| Markt_651724-09-3_2 | active |  | 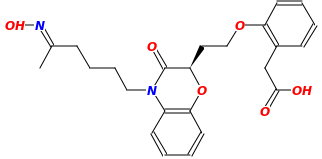 |
| Markt_853652-40-1_1 | active |  | 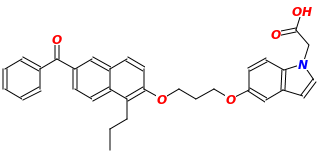 |
| Markt_853652-40-1_2 | active |  | 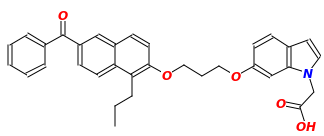 |
| Markt_BRL48482_1 | active |  | 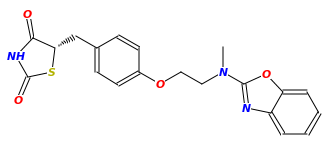 |
| Markt_BRL48482_2 | active |  | 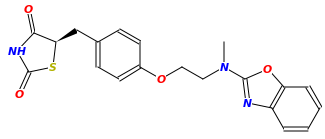 |
| Markt_BVT13 | active |  | 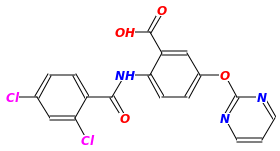 |
| Markt_CLX-M1_1 | active |  | 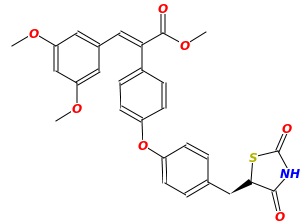 |
| Markt_CLX-M1_2 | active |  | 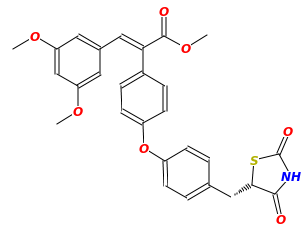 |
| Markt_KRP297_1 | active |  | 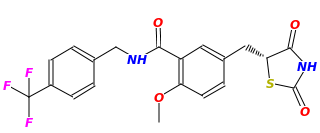 |
| Markt_KRP297_2 | active |  | 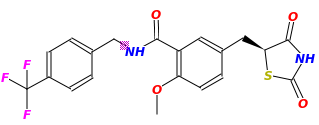 |
| Markt_NNC61-4424_1 | active |  | 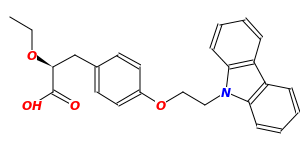 |
| Markt_NNC61-4424_2 | active |  | 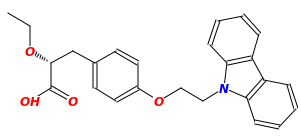 |
| Tesaglitazar | active |  | 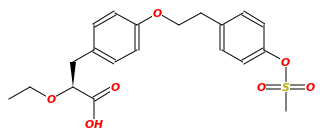 |
| Troglitazone_1 | active |  | 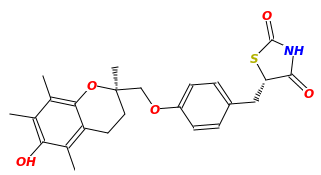 |
| Troglitazone_2 | active |  | 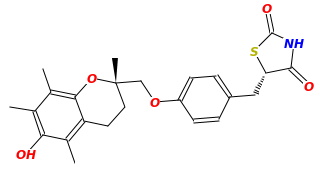 |
| Troglitazone_3 | active |  | 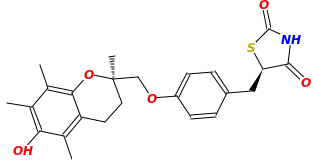 |
| Troglitazone_4 | active |  | 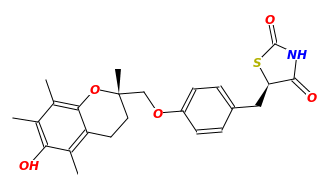 |
| ψ-baptigenin | active |  | 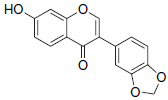 |
| Punicic Acid | active |  |  |
| Tanrikulu1 | active |  | 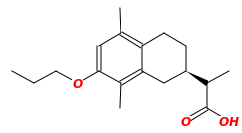 |
| Tanrikulu2_1 | active |  | 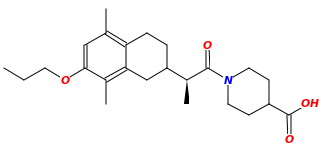 |
| Tanrikulu2_2 | active |  | 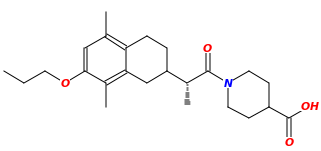 |
| Tanrikulu2_3 | active |  | 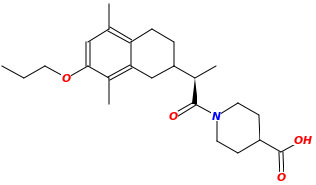 |
| Tanrikulu2_4 | active |  | 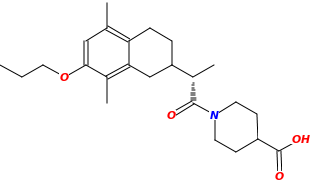 |
| Tanrikulu3_1 | inactive |  | 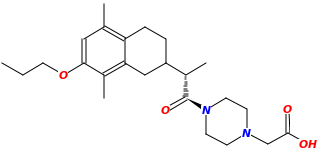 |
| Tanrikulu3_2 | inactive |  | 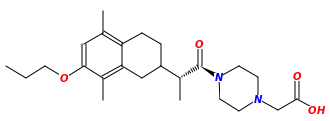 |
| Tanrikulu3_3 | inactive |  | 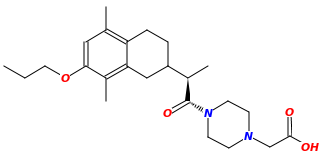 |
| Tanrikulu3_4 | inactive |  | 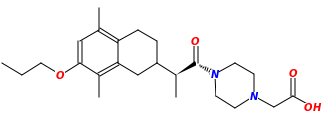 |
| Tanrikulu4 | inactive |  | 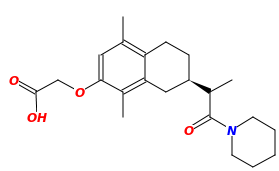 |
| Tanrikulu5 | inactive |  | 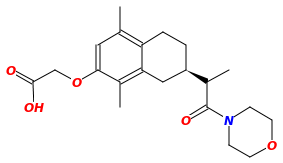 |
| Tanrikulu6 | inactive |  | 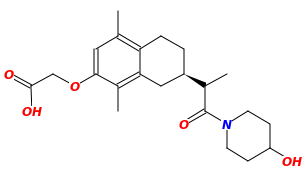 |
| Tanrikulu7_1 | inactive |  | 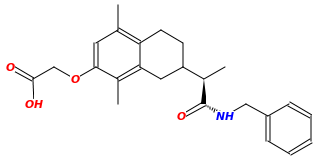 |
| Tanrikulu7_2 | inactive |  | 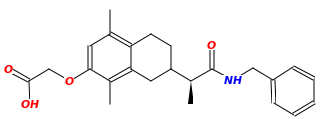 |
| Tanrikulu7_3 | inactive |  | 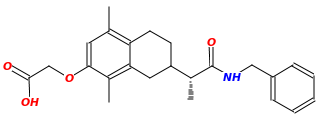 |
| Tanrikulu7_4 | inactive |  | 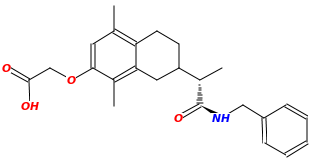 |
| Tanrikulu8_1 | inactive |  | 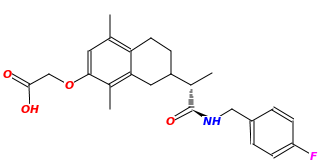 |
| Tanrikulu8_2 | inactive |  | 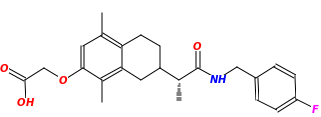 |
| Tanrikulu8_3 | inactive |  | 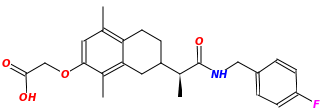 |
| Tanrikulu8_4 | inactive |  | 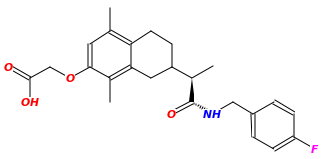 |
| α-EleostearicAcid | active |  | 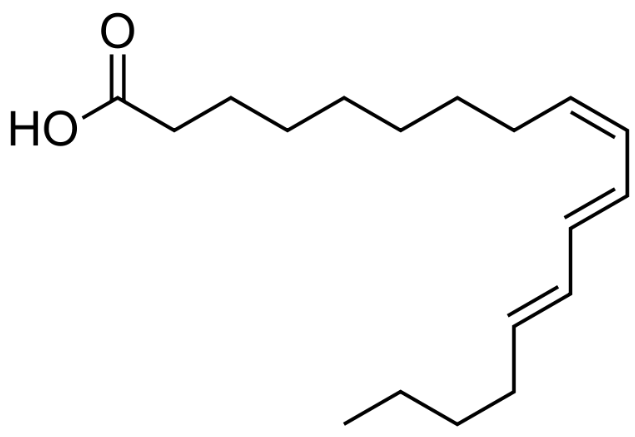 |
| Apigenin | active |  | 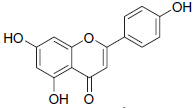 |
| β-EleostearicAcid | low active |  | 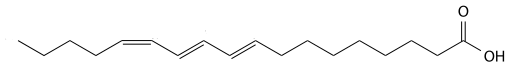 |
| Biochanin-A | low active |  | 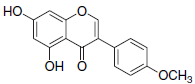 |
| Chrysin | low active |  | 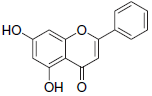 |
| Dihydroquercetin | inactive |  | 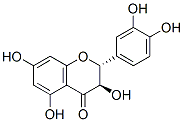 |
| Genistein | low active |  | 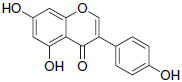 |
| Hesperidin | low active |  | 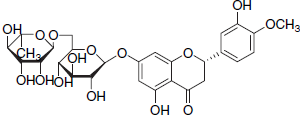 |
| Omega-3 conjugated linoleic acid | inactive |  | 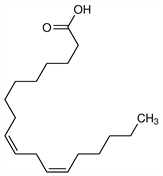 |
| Rosiglitazone | active |  | 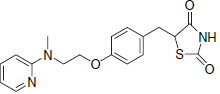 |
| Vitexin | inactive |  | 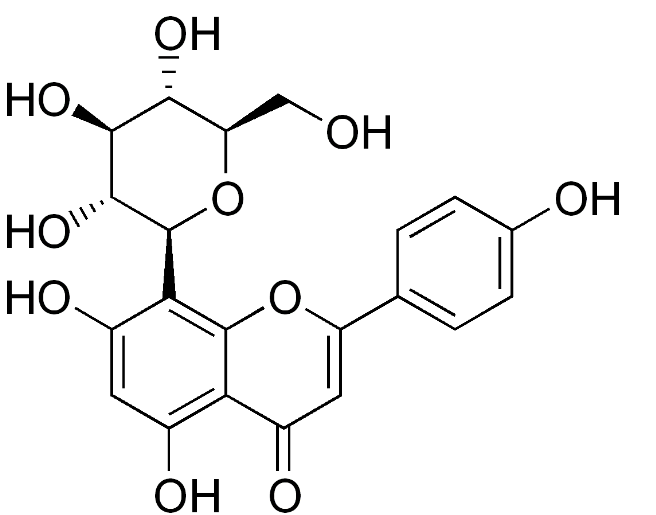 |
