# Supplementary material for: Dietary α-Eleostearic Acid Ameliorates Experimental Inflammatory Bowel Disease in Mice by Activating Peroxisome Proliferator-Activated Receptor-γ
Source: PLoS One. 2011 Aug 31;6(8):e24031. doi: 10.1371/journal.pone.0024031 (PMC3164124; doi:10.1371/journal.pone.0024031)
Supplement: Table S4 — List of atoms for key residues common to rosiglitazone- and fatty acid-containing PDB structures used to assess potential interactions between docked poses and the protein structure model. (DOC) [file pone.0024031.s005.doc]

**Table S4** List of atoms for key residues common to rosiglitazone- and fatty acid-containing PDB structures used to assess potential interactions between docked poses and the protein structure model.

| **Residue Name** | **Residue Number** | **Atom Name**^1^ |
| --- | --- | --- |
| ILE | 281 | CG2 |
| SER | 289 | CB |
| SER | 289 | OG |
| HIS | 323 | NE2 |
| ILE | 326 | CG2 |
| LEU | 330 | CD1 |
| LEU | 330 | CD2 |
| ILE | 341 | CG2 |
| MET | 348 | CE |
| MET | 364 | CE |
| LYS | 367 | CE |
| HIS | 449 | CE1 |
| HIS | 449 | NE2 |
| TYR | 473 | OH* |

^1^Atom names are represented by two or three characters, where the first is the atom type, the second is the position relative to the residue structure, and the number of the atom is more than one is as the designated position (e.g., CG2 corresponds to the second carbon atom in the gamma position).

*Hydroxyl oxygen.
