# Supplementary material for: Dietary α-Eleostearic Acid Ameliorates Experimental Inflammatory Bowel Disease in Mice by Activating Peroxisome Proliferator-Activated Receptor-γ
Source: PLoS One. 2011 Aug 31;6(8):e24031. doi: 10.1371/journal.pone.0024031 (PMC3164124; doi:10.1371/journal.pone.0024031)
Supplement: Table S5 — Predicted hydrophobic and hydrogen bond interactions for ligands in cross-docking test set relative to a reference list of interactions common to rosiglitazone and selected fatty acids. Poses were taken from docking of each ligand into each of the three listed PPARγ PDB files (top row). Ligand IDs refer to compounds listed in Table 3. (DOC) [file pone.0024031.s006.doc]

**Table S5** Predicted hydrophobic and hydrogen bond interactions for ligands in cross-docking test set relative to a reference list of interactions common to rosiglitazone and selected fatty acids. Poses were taken from docking of each ligand into each of the three listed PPARγ PDB files (top row). Ligand IDs refer to compounds listed in Table 3.

|  | **1FM6** | | **1ZGY** | | **2PRG** | |
| --- | --- | --- | --- | --- | --- | --- |
| **Ligand ID** | Hydrophobic | Hydrogen bond | Hydrophobic | Hydrogen bond | Hydrophobic | Hydrogen bond |
| 243 | 8 | 2 | 10 | 1 | 6 | 4 |
|  | 6 | 0 | 5 | 4 | 8 | 4 |
|  | 3 | 1 | 11 | 2 | 9 | 5 |
| 4hd | 9 | 3 | 8 | 5 | 12 | 2 |
|  | 14 | 1 | 10 | 5 | 13 | 0 |
|  | 15 | 3 | 9 | 3 | 16 | 0 |
| 570 | 11 | 0 | 12 | 1 | 5 | 0 |
|  | 9 | 0 | 11 | 1 | 6 | 0 |
|  | 9 | 0 | 7 | 1 | 5 | 0 |
| 9ho | 9 | 4 | 9 | 3 | 9 | 4 |
|  | 8 | 5 | 15 | 6 | 10 | 4 |
|  | 7 | 5 | 10 | 6 | 5 | 4 |
| drh | 7 | 0 | 14 | 6 | 5 | 4 |
|  | 9 | 0 | 12 | 3 | 7 | 0 |
|  | 9 | 0 | 13 | 3 | 8 | 4 |
| drj | 9 | 0 | 12 | 4 | 12 | 5 |
|  | 13 | 4 | 13 | 4 | 10 | 5 |
|  | 13 | 4 | 9 | 4 | 12 | 4 |
| dry | 12 | 0 | 7 | 5 | 11 | 3 |
|  | 9 | 0 | 6 | 3 | 9 | 4 |
|  | 13 | 0 | 8 | 3 | 8 | 3 |
| eha | 10 | 0 | 6 | 2 | 10 | 3 |
|  | 10 | 2 | 6 | 3 | 9 | 4 |
|  | 9 | 2 | 14 | 0 | 11 | 4 |
| et1 | 5 | 0 | 8 | 1 | 6 | 1 |
|  | 5 | 0 | 8 | 1 | 7 | 1 |
|  | 4 | 0 | 8 | 1 | 7 | 1 |
| hxa | 14 | 3 | 10 | 5 | 13 | 4 |
|  | 6 | 0 | 10 | 3 | 10 | 2 |
|  | 7 | 0 | 8 | 3 | 14 | 2 |
| ptg1 | 16 | 5 | 13 | 6 | 15 | 5 |
|  | 16 | 5 | 13 | 6 | 14 | 2 |
|  | 16 | 5 | 11 | 6 | 15 | 4 |
| ptg2 | 6 | 2 | 11 | 1 | 16 | 4 |
|  | 5 | 2 | 10 | 1 | 16 | 5 |
|  | 9 | 5 | 11 | 1 | 11 | 3 |
