# Supplementary material for: Dietary α-Eleostearic Acid Ameliorates Experimental Inflammatory Bowel Disease in Mice by Activating Peroxisome Proliferator-Activated Receptor-γ
Source: PLoS One. 2011 Aug 31;6(8):e24031. doi: 10.1371/journal.pone.0024031 (PMC3164124; doi:10.1371/journal.pone.0024031)
Supplement: Table S6 — Presence or absence of potential hydrogen bond interactions between indicated residues of selected protein structure models and replicate poses of ligands listed by ID. A single “x” indicates one potential interaction for the listed residue was found for the specified ligand, whereas more than one “x” indicates more than one interaction (e.g., “xx” indicates two interactions found). (N = 3) (DOC) [file pone.0024031.s007.doc]

**Table S6** Presence or absence of potential hydrogen bond interactions between indicated residues of selected protein structure models and replicate poses of ligands listed by ID. A single “x” indicates one potential interaction for the listed residue was found for the specified ligand, whereas more than one “x” indicates more than one interaction (e.g., “xx” indicates two interactions found). (N = 3)

|  | **1FM6** | | | | **1ZGY** | | | | **2PRG** | | | |
| --- | --- | --- | --- | --- | --- | --- | --- | --- | --- | --- | --- | --- |
| **Ligand ID** | HIS 323 NE2 | HIS 449 NE2 | SER 289 OG | TYR 473 OH | HIS 323 NE2 | HIS 449 NE2 | SER 289 OG | TYR 473 OH | HIS 323 NE2 | HIS 449 NE2 | SER 289 OG | TYR 473 OH |
| 243 |  | x |  | x |  | x |  |  | x | x | x | x |
|  |  |  |  |  |  | x | xx | x | x | x | x | x |
|  |  |  |  | x |  | x |  | x | x | x | x | xx |
| 4hd |  | xx |  | x | x | x | x | xx | x |  | x |  |
|  |  | x |  |  | x | x | x | xx |  |  |  |  |
|  | x |  | x | x | x |  | x | x |  |  |  |  |
| 570 |  |  |  |  |  |  | x |  |  |  |  |  |
|  |  |  |  |  |  |  | x |  |  |  |  |  |
|  |  |  |  |  |  |  | x |  |  |  |  |  |
| 9ho | x |  | x | xx |  | x | x | x | x | x | x | x |
|  | x | x | x | xx | x | x | xx | xx | x | x | x | x |
|  | x | x | x | xx | x | x | xx | xx | x | x | x | x |
| drh |  |  |  |  | x | x | xx | xx | x | x | x | x |
|  |  |  |  |  |  | xx |  | x |  |  |  |  |
|  |  |  |  |  |  | xx |  | x | x | x | x | x |
| drj |  |  |  |  | x | x | x | x | x | xx |  | xx |
|  | x | x | x | x | x | x | x | x | x | xx |  | xx |
|  | x | x | x | x | x | x | x | x | x | x | x | x |
| dry |  |  |  |  | x | x | x | xx | x | x |  | x |
|  |  |  |  |  |  | x | x | x | x | x | x | x |
|  |  |  |  |  |  | x | x | x | x | x |  | x |
| eha |  |  |  |  |  | x |  | x |  | x | x | x |
|  | x |  |  | x |  | x | x | x | x | x |  | xx |
|  |  | x |  | x |  |  |  |  | x | x |  | xx |
| et1 |  |  |  |  |  |  | x |  |  |  | x |  |
|  |  |  |  |  |  |  | x |  |  |  | x |  |
|  |  |  |  |  |  |  | x |  |  |  | x |  |
| hxa |  | x | x | x | x | x | x | xx |  | xx |  | xx |
|  |  |  |  |  | x |  | x | x | x |  | x |  |
|  |  |  |  |  | x |  | x | x | x |  | x |  |
| ptg1 | x | x | x | xx | x | x | xx | xx | x | x | x | xx |
|  | x | x | x | xx | x | x | xx | xx | x |  | x |  |
|  | x | x | x | xx | x | x | xx | xx | x | x | x | x |
| ptg2 |  | x | x |  |  |  | x |  | x | x | x | x |
|  |  | x | x |  |  |  | x |  | x | x | x | xx |
|  | x | x | x | xx |  |  | x |  | x | x | x |  |
