# Supplementary material for: Dietary α-Eleostearic Acid Ameliorates Experimental Inflammatory Bowel Disease in Mice by Activating Peroxisome Proliferator-Activated Receptor-γ
Source: PLoS One. 2011 Aug 31;6(8):e24031. doi: 10.1371/journal.pone.0024031 (PMC3164124; doi:10.1371/journal.pone.0024031)
Supplement: Table S7 — Predicted hydrophobic and hydrogen bond interactions for ligands in small-scale screening test set relative to a reference list of interactions common to rosiglitazone and selected fatty acids (Table S4). Poses were taken from docking of each ligand into each of the three listed PPARγ PDB files (top row). Predicted free energy of binding is listed as kcal/mol. (DOC) [file pone.0024031.s008.doc]

**Table S7** Predicted hydrophobic and hydrogen bond interactions for ligands in small-scale screening test set relative to a reference list of interactions common to rosiglitazone and selected fatty acids (Table S3). Poses were taken from docking of each ligand into each of the three listed PPARγ PDB files (top row). Predicted free energy of binding is listed as kcal/mol.

|  | **1FM6** | | | **1ZGY** | | | **2PRG** | | |
| --- | --- | --- | --- | --- | --- | --- | --- | --- | --- |
| **Ligand** | kcal/mol | Hydrophobic | Hydrogen Bonds | kcal/mol | Hydrophobic | Hydrogen Bonds | kcal/mol | Hydrophobic | Hydrogen Bonds |
| Farglitazar | -10.4 | 10 | 0 | -11.2 | 10 | 0 | -10.5 | 5 | 0 |
| Indol-1-yl acetic acid | -10.1 | 8 | 2 | -10.4 | 8 | 2 | -10.6 | 9 | 4 |
| 5-substituted indoleoxyacetic acid analogue | -10.1 | 9 | 0 | -10.4 | 9 | 0 | -10.4 | 10 | 3 |
| (2R)-ureidofibrate-like | -8.7 | 14 | 4 | -9.0 | 14 | 4 | -8.3 | 10 | 5 |
| (2S)-ureidofibrate-like | -8.2 | 10 | 0 | -8.9 | 10 | 0 | -8.3 | 6 | 4 |
| 9-HODE | -6.5 | 9 | 5 | -6.6 | 9 | 5 | -6.6 | 8 | 4 |
| 13-HODE | -7.0 | 6 | 2 | -6.6 | 6 | 2 | -6.3 | 7 | 4 |
| Indeglitazar | -8.1 | 4 | 0 | -8.5 | 4 | 0 | -8.5 | 6 | 1 |
| α-Eleostearic Acid | -6.4 | 8 | 5 | -6.5 | 8 | 5 | -6.5 | 6 | 4 |
| apigenin | -8.1 | 0 | 0 | -8.1 | 0 | 0 | -7.8 | 11 | 2 |
| β-Eleostearic Acid | -6.1 | 10 | 5 | -6.2 | 10 | 5 | -6.0 | 13 | 5 |
| BiochaninA | -8.0 | 0 | 0 | -7.7 | 0 | 0 | -7.8 | 2 | 0 |
| Calendic Acid | -6.1 | 10 | 5 | -5.8 | 10 | 5 | -5.7 | 11 | 4 |
| Catalpic Acid | -5.9 | 8 | 4 | -5.9 | 8 | 4 | -6.0 | 8 | 2 |
| chrysin | -7.8 | 6 | 0 | -8.4 | 6 | 0 | -7.8 | 0 | 0 |
| CLA (18C:c9,t11) | -6.0 | 9 | 1 | -6.4 | 9 | 1 | -6.3 | 8 | 4 |
| Dihydroquercetin | -8.5 | 3 | 0 | -8.0 | 3 | 0 | -7.7 | 6 | 2 |
| Genistein | -8.1 | 0 | 0 | -7.5 | 0 | 0 | -8.0 | 4 | 0 |
| Hesperidin | -9.2 | 6 | 0 | -10.0 | 6 | 0 | -9.2 | 9 | 0 |
| Jacaric Acid | -5.9 | 13 | 5 | -6.2 | 13 | 5 | -5.8 | 7 | 4 |
| Kuroda_No10 | -7.1 | 9 | 0 | -7.3 | 9 | 0 | -7.2 | 8 | 0 |
| Kuroda_No15 | -7.3 | 2 | 0 | -7.5 | 2 | 0 | -7.5 | 10 | 3 |
| Kuroda_No16 | -7.7 | 0 | 0 | -8.7 | 0 | 0 | -8.9 | 6 | 0 |
| Kuroda_No2 | -8.4 | 6 | 1 | -8.8 | 6 | 1 | -9.2 | 0 | 0 |
| Kuroda_No3 | -8.7 | 3 | 0 | -9.2 | 3 | 0 | -9.0 | 6 | 0 |
| Kuroda_No34 | -9.8 | 4 | 0 | -9.9 | 4 | 0 | -9.2 | 4 | 0 |
| Kuroda_No38 | -8.9 | 0 | 0 | -9.4 | 0 | 0 | -9.5 | 9 | 1 |
| Kuroda_No39 | -8.4 | 12 | 0 | -8.1 | 12 | 0 | -8.6 | 10 | 3 |
| Kuroda_No5 | -7.5 | 6 | 0 | -9.2 | 6 | 0 | -8.7 | 8 | 0 |
| Kuroda_No6 | -7.5 | 8 | 0 | -9.0 | 8 | 0 | -8.1 | 11 | 0 |
| Markt_  264908-13-6_1 | -9.9 | 8 | 3 | -10.5 | 8 | 3 | -10.1 | 11 | 2 |
| Markt_  264908-13-6_2 | -10.4 | 6 | 4 | -9.6 | 6 | 4 | -10.3 | 5 | 4 |
| Markt_  264908-13-6_3 | -10.1 | 14 | 3 | -10.0 | 14 | 3 | -9.8 | 13 | 0 |
| Markt_  264908-13-6_4 | -9.5 | 2 | 0 | -8.8 | 2 | 0 | -9.9 | 4 | 2 |
| Markt_  651724-09-3_1 | -8.3 | 5 | 0 | -8.7 | 5 | 0 | -8.4 | 7 | 0 |
| Markt_  651724-09-3_2 | -8.6 | 5 | 0 | -8.8 | 5 | 0 | -8.7 | 14 | 2 |
| Markt_  853652-40-1_1 | -10.2 | 9 | 2 | -10.3 | 9 | 2 | -10.7 | 9 | 4 |
| Markt_  853652-40-1_2 | -10.2 | 14 | 5 | -10.6 | 14 | 5 | -10.7 | 11 | 4 |
| Markt_  BRL48482_1 | -8.9 | 10 | 1 | -9.3 | 10 | 1 | -8.9 | 7 | 2 |
| Markt_  BRL48482_2 | -9.1 | 7 | 4 | -9.3 | 7 | 4 | -9.5 | 12 | 4 |
| Markt_BVT13 | -8.1 | 4 | 0 | -8.6 | 4 | 0 | -8.2 | 8 | 0 |
| Markt_  CLX-M1_1 | -9.9 | 10 | 4 | -10.2 | 10 | 4 | -9.9 | 13 | 4 |
| Markt_  CLX-M1_2 | -8.4 | 6 | 0 | -8.6 | 6 | 0 | -8.7 | 3 | 0 |
| Markt_  KRP297_1 | -9.2 | 9 | 0 | -9.3 | 9 | 0 | -9.2 | 7 | 2 |
| Markt_  KRP297_2 | -9.4 | 6 | 1 | -9.3 | 6 | 1 | -9.3 | 12 | 4 |
| Markt_  NNC61-4424_1 | -9.5 | 14 | 5 | -9.4 | 14 | 5 | -9.8 | 6 | 4 |
| Markt_  NNC61-4424_2 | -9.2 | 10 | 5 | -8.2 | 10 | 5 | -9.3 | 12 | 2 |
| Markt_  tesaglitazar | -8.7 | 4 | 5 | -8.6 | 4 | 5 | -8.6 | 6 | 4 |
| Markt_  troglitazone_1 | -10.2 | 3 | 4 | -10.4 | 3 | 4 | -10.5 | 8 | 4 |
| Markt_  troglitazone_2 | -9.9 | 4 | 4 | -10.1 | 4 | 4 | -10.6 | 10 | 4 |
| Markt_  troglitazone_3 | -9.5 | 5 | 0 | -9.9 | 5 | 0 | -9.9 | 5 | 2 |
| Markt_  troglitazone_4 | -9.2 | 5 | 0 | -9.7 | 5 | 0 | -9.7 | 5 | 2 |
| Omega-3 | -6.6 | 11 | 5 | -6.5 | 11 | 5 | -6.7 | 9 | 4 |
| Ψ-Baptigenin | -8.1 | 4 | 0 | -8.2 | 4 | 0 | -8.5 | 8 | 2 |
| Punicic Acid | -6.3 | 10 | 4 | -5.7 | 10 | 4 | -6.2 | 11 | 3 |
| Rosiglitazone | -8.7 | 6 | 4 | -8.5 | 6 | 4 | -8.7 | 9 | 4 |
| Tanrikulu1 | -7.6 | 9 | 0 | -7.5 | 9 | 0 | -7.3 | 7 | 0 |
| Tanrikulu2_1 | -9.3 | 4 | 0 | -8.7 | 4 | 0 | -8.8 | 3 | 0 |
| Tanrikulu2_2 | -8.2 | 13 | 0 | -9.0 | 13 | 0 | -8.7 | 8 | 0 |
| Tanrikulu2_3 | -9.6 | 10 | 1 | -9.4 | 10 | 1 | -8.8 | 3 | 0 |
| Tanrikulu2_4 | -8.8 | 8 | 0 | -9.1 | 8 | 0 | -8.8 | 4 | 0 |
| Tanrikulu3_1 | -9.2 | 7 | 0 | -8.8 | 7 | 0 | -8.8 | 6 | 0 |
| Tanrikulu3_2 | -9.0 | 10 | 0 | -9.0 | 10 | 0 | -8.7 | 10 | 0 |
| Tanrikulu3_3 | -8.8 | 6 | 1 | -8.5 | 6 | 1 | -8.7 | 8 | 1 |
| Tanrikulu3_4 | -9.1 | 6 | 0 | -8.5 | 6 | 0 | -8.5 | 10 | 1 |
| Tanrikulu4 | -8.4 | 4 | 0 | -8.8 | 4 | 0 | -9.4 | 8 | 0 |
| Tanrikulu5 | -8.1 | 4 | 0 | -9.2 | 4 | 0 | -9.4 | 8 | 1 |
| Tanrikulu6 | -8.7 | 3 | 0 | -8.4 | 3 | 0 | -8.8 | 4 | 0 |
| Tanrikulu7_1 | -9.6 | 7 | 0 | -9.5 | 7 | 0 | -9.1 | 4 | 0 |
| Tanrikulu7_2 | -9.1 | 6 | 0 | -9.0 | 6 | 0 | -9.1 | 10 | 4 |
| Tanrikulu7_3 | -9.1 | 7 | 0 | -9.4 | 7 | 0 | -9.1 | 3 | 0 |
| Tanrikulu7_4 | -9.2 | 8 | 0 | -9.6 | 8 | 0 | -9.1 | 5 | 0 |
| Tanrikulu8_1 | -9.5 | 9 | 1 | -9.8 | 9 | 1 | -9.2 | 7 | 0 |
| Tanrikulu8_2 | -9.3 | 9 | 0 | -9.4 | 9 | 0 | -9.5 | 11 | 4 |
| Tanrikulu8_3 | -9.6 | 4 | 0 | -9.4 | 4 | 0 | -9.2 | 12 | 3 |
| Tanrikulu8_4 | -9.5 | 8 | 0 | -9.8 | 8 | 0 | -9.4 | 12 | 5 |
| Vitexin | -9.1 | 4 | 0 | -11.2 | 4 | 0 | -8.6 | 7 | 0 |
