# Supplementary material for: Dietary α-Eleostearic Acid Ameliorates Experimental Inflammatory Bowel Disease in Mice by Activating Peroxisome Proliferator-Activated Receptor-γ
Source: PLoS One. 2011 Aug 31;6(8):e24031. doi: 10.1371/journal.pone.0024031 (PMC3164124; doi:10.1371/journal.pone.0024031)
Supplement: Table S9 — Predicted free energy of binding and interaction counts for conjugated trienes. Docking was performed using AD4 with three top-binding replicates for each ligand (150 total conformations). The highest energy conformation with the highest number of hydrogen bonds was used for analysis in Table 4. (DOC) [file pone.0024031.s010.doc]

**Table S9** Predicted free energy of binding and interaction counts for conjugated trienes. Docking was performed using AD4 with three top-binding replicates for each ligand (150 total conformations). The highest energy conformation with the highest number of hydrogen bonds was used for analysis in Table 4.

| **Ligand** | **kcal/mol** | **Hydrogen bond** | **Hydrophobic** |
| --- | --- | --- | --- |
| α-Eleostearic | -5.75 | 2 | 15 |
|  | -5.73 | 1 | 15 |
|  | -5.6 | 3 | 16 |
| Calendic | -4.49 | 0 | 29 |
|  | -3.95 | 1 | 13 |
|  | -4.47 | 2 | 7 |
| Catalpic | -4.72 | 1 | 13 |
|  | -4.48 | 3 | 9 |
|  | -4.31 | 1 | 23 |
| Jacaric | -4.81 | 2 | 11 |
|  | -4.97 | 1 | 9 |
|  | -4.5 | 3 | 16 |
| Punicic | -4.3 | 2 | 16 |
|  | -4.28 | 3 | 15 |
|  | -3.78 | 1 | 10 |
