# Supplementary material for: Dietary α-Eleostearic Acid Ameliorates Experimental Inflammatory Bowel Disease in Mice by Activating Peroxisome Proliferator-Activated Receptor-γ
Source: PLoS One. 2011 Aug 31;6(8):e24031. doi: 10.1371/journal.pone.0024031 (PMC3164124; doi:10.1371/journal.pone.0024031)
Supplement: Formulas S1 — (DOC) [file pone.0024031.s011.doc]

**Formulas**

Standard Deviation (*σ*) refers to the degree to which the measured values deviate from the mean.


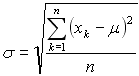
, where *x* is a single value, *k* is the index for the value, *μ* is the population mean, and *n* is the number of values. Variance defines how far the measured values are from each other and is defined as *σ^2^*.

Root Mean-Squared Deviation (RMSD) is a measure of the change in geometry and orientation of a pose from that of the reference control structure.

RMSD
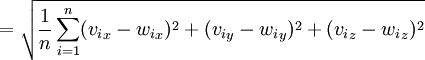
, where *n* number of equivalent pairs of atoms, and *v* and *w* are the cooridinate sets for the pose and reference structures.
